# Moderate DNA methylation changes associated with nitrogen remobilization and leaf senescence in Arabidopsis

**DOI:** 10.1093/jxb/erac167

**Published:** 2022-05-13

**Authors:** Emil Vatov, Ulrike Zentgraf, Uwe Ludewig

**Affiliations:** Institute of Crop Science, Nutritional Crop Physiology, University of Hohenheim, Stuttgart, D-70593, Germany; Center for Molecular Biology of Plants (ZMBP), University of Tübingen, Tübingen, D-72076, Germany; Center for Molecular Biology of Plants (ZMBP), University of Tübingen, Tübingen, D-72076, Germany; Institute of Crop Science, Nutritional Crop Physiology, University of Hohenheim, Stuttgart, D-70593, Germany; University of Auckland, New Zealand

**Keywords:** Epigenetics, flowering, longevity, methylome, nutrition, phytohormones, whole genome bisulfite sequencing

## Abstract

The lifespan of plants is restricted by environmental and genetic components. Following the transition to reproductive growth, leaf senescence ends cellular life in monocarpic plants to remobilize nutrients to storage organs. In Arabidopsis, we initially observed altered leaf to seed ratios, faster senescence progression, altered leaf nitrogen recovery after transient nitrogen removal, and ultimately enhanced nitrogen remobilization from the leaves in two methylation mutants (*ros1* and the triple *dmr1/2 cmt3* knockout). Analysis of the DNA methylome in wild type Col-0 leaves identified an initial moderate decline of cytosine methylation with progressing leaf senescence, predominantly in the CG context. Late senescence was associated with moderate *de novo* methylation of cytosines, primarily in the CHH context. Relatively few differentially methylated regions, including one in the *ROS1* promoter linked to down-regulation of *ROS1*, were present, but these were unrelated to known senescence-associated genes. Differential methylation patterns were identified in transcription factor binding sites, such as the W-boxes that are targeted by WRKYs. Methylation in artificial binding sites impaired transcription factor binding *in vitro*. However, it remains unclear how moderate methylome changes during leaf senescence are linked with up-regulated genes during senescence.

## Teaser

Arabidopsis mutants defective in DNA methylation have altered senescence, and leaf methylation patterns moderately change during ageing, but there is little relation to senescence-associated genes.

## Introduction

Senescence in monocarpic plants, such as Arabidopsis, is a developmentally regulated process that leads to the controlled death of the whole organism, except for the dormant seeds. After the transition to reproductive growth, not only the fully expanded leaves, but all leaves undergo senescence processes and are turned into source leaves for the newly developing flowers and seeds. The systematic dismantling of the chloroplasts is characteristic of leaf senescence, resulting in a visible loss of green coloration. Rubisco, which is involved in photosynthesis and located in the chloroplast stroma, is the most abundant protein in leaf tissue and therefore accounts for a large proportion of the nitrogen within a leaf. During senescence, nitrogen (N) remobilization is thus coupled to chloroplast dismantling; the degradation of photosynthesis-related proteins results in reduced photosynthetic capacity (Diaz *et al.*, 2008; Masclaux-Daubresse *et al.*, 2010; Havé *et al.*, 2017). Even though this compromises carbohydrate assimilation, soluble sugars and hexoses with signalling roles transiently increase in leaves during senescence (Wingler *et al.*, 2006).

In addition to their deteriorative power, reactive oxygen species (ROS), and in particular hydrogen peroxide (H_2_O_2_), increase during the transition to reproductive growth and, in turn, correlate with the expression of many senescence-associated genes including transcriptional regulators. The main transcription factor families orchestrating the process are NAC, WRKY, C2H2, and MYB (Breeze *et al.*, 2011; Liebsch and Keech, 2016). WRKY53 appears to play a key role in early senescence regulation and is tightly controlled by many different mechanisms at the transcriptional and post-transcriptional level (Zentgraf and Doll, 2019). As almost all WRKY factor genes carry binding sites for other WRKY factors in their promoters (Dong *et al.*, 2003), WRKY factors regulate each other in complex regulatory networks. For example, WRKY18 has been characterized as an upstream negative regulator, downstream target, and protein interaction partner of WRKY53. Moreover, WKRY25 influences the expression of *WRKY53* and *WRKY18* in a redox-dependent manner but is itself also involved in regulating intracellular hydrogen peroxide concentrations (Doll *et al.*, 2020). Moreover, WRKY53 and WRKY18 have been identified as regulators of the sugar response gene *GPT2* via the recruitment of HAC1 (Chen *et al.*, 2019).

The involvement of epigenetic mechanisms, in the form of active histone modifications related to chromatin decondensation and the regulation of key senescence regulators, e.g. *WRKY53*, has also been observed (Ay *et al.*, 2009; Brusslan *et al.*, 2012). The expression of the *WRKY53* locus itself is under epigenetic control and the WRKY53 protein participates in the epigenetic control of other senescence regulators, i.e. the SANT-domain-containing protein POWERDRESS recruits the histone deacetylase HDA9 to the W-box-containing promoter regions of negative senescence regulator genes (e.g. *WRKY57*, *APG9*, and *NPX1*) with the help of WRKY53 (Chen *et al.*, 2016).

Whereas ageing in humans is associated with genome-wide hypomethylation and some locus-specific hypermethylation (Jones *et al.*, 2015), initial studies on cytosine methylation changes during leaf senescence and ageing had revealed contradictory evidence. In a variety of plant species and under different experimental conditions, cytosine methylation has been found to either increase or decrease (Dubrovina and Kiselev, 2016). Cytosine methylation is most important for transposon silencing, but also regulates gene expression, histone modifications, and heterochromatin formation (Sequeira-Mendes *et al.*, 2014; Lei *et al.*, 2015). In plants, cytosine methylation is found in CG, CHG, and CHH contexts, where H stands for nucleotides other than G (Stroud *et al.*, 2013). In Arabidopsis, methylation of some targets tended to be reduced during growth and ageing and this was correlated with reduced expression of the methyltransferase genes *MET1* (acting predominantly on CG) and *CMT3* (acting on CHG motifs), indicating less maintenance methylation (Ogneva *et al.*, 2016). DNA demethylation can be either passive or active (Ikeda and Kinoshita, 2009). During DNA replication, the lack of maintenance can cause the passive loss of established methylation patterns, whereas active demethylation occurs enzymatically via base excision and repair by REPRESSOR OF SILENCING 1 (ROS1), DME, DML2, and DML3. ROS1 is particularly important for counteracting DNA methylation established by the RNA-directed DNA methylation (RdDM) pathway (Gong *et al.*, 2002; Zhang *et al.*, 2018). RdDM targets *de novo* methylation in all cytosine contexts and is triggered by the recruitment of DRM2 (reviewed by Matzke *et al.*, 2015).

Detailed whole genome bisulfite sequencing (WGBS) on leaves during dark-induced leaf senescence in Arabidopsis revealed that the methylation landscape remains largely stable, with some hypomethylation occurring in CHH contexts (Trejo-Arellano *et al.*, 2020). By contrast, Yuan *et al.* (2020) identified a genome wide reduction in methylation in all three cytosine contexts in age-related senescence, with a particular reduction of CG methylation in CG-rich sequences in close proximity to gene transcription start sites (TSS). Furthermore, the comparison with *dml3* knockout mutants identified a substantial correlation between cytosine methylation in the genes and their expression, suggesting an important role of the DML3-induced demethylation of gene regulatory regions during senescence (Yuan *et al.*, 2020). However, despite clear evidence that methylation affects gene expression of some individual genes, a causal general relationship between global gene methylation patterns and transcript abundance is lacking (Ay *et al.*, 2014; Quadrana and Colot, 2016; Zhang *et al.*, 2018). Besides, natural age-related senescence and stress-induced premature senescence are known to exhibit major differences in, for example, their execution signalling and their transcriptome profiles (Buchanan-Wollaston *et al.*, 2005). This may account for differences in methylation patterns in previous different experimental settings. Moreover, He *et al.* (2018) discovered a naturally occurring epiallele involved in the regulation of leaf senescence, additionally implying that cytosine methylation plays a role in senescence and in the adaptation of plants to local climates. Furthermore, progressive DNA demethylation occurs during tomato fruit ripening, with a profound effect on the fruit transcriptome (Lang *et al.*, 2017). Finally, cytosine methylation regulates key genetic loci involved in flower induction: *FLOWERING LOCUS C* (*FLC*; Finnegan *et al.*, 2005), *FLOWERING TIME* (*FT*; Zicola *et al.*, 2019) and *FLOWERING WAGENINGEN* (*FWA*; Kinoshita *et al.*, 2007). Although the key causal molecular link between flowering and the initiation of senescence has not yet been identified, cytosine methylation may play a role with its impact on flowering time and therefore affect the initiation of senescence. In addition to biotic stress (Dowen *et al.*, 2012) and other, abiotic stresses (Zhang *et al.*, 2018), nutrient deficiencies elicit changes in the methylomes of Arabidopsis (Yong-Villalobos *et al.*, 2015; Chen *et al.*, 2018), maize (Mager and Ludewig, 2018), and rice (Kou *et al.*, 2011). Thus, changes in the methylation landscape as a result of nutrient deficiency could (indirectly) have an impact on flowering time, senescence, and nutrient remobilization.

In the present study, we analysed whether mutants that are defective in proper methylation exhibit altered development at the ultimate vegetative stages and during the generative growth phase. We employed the Arabidopsis hypomethylated triple mutant *drm1 drm2 cmt3* (*ddc*) and the hypermethylated *ros1* mutant in the genetic background of Col-0. We hypothesized that specific alterations of the methylation landscape are associated with the progression of generative growth and leaf senescence and performed WGBS at four time points, starting at the transition of the shoot apical meristem into reproductive growth. We further tested whether this developmental progress destabilizes the methylome and results in more variation. Our physiological, bioinformatic, and biochemical analyses are consistent with moderately distinct cytosine methylation patterns at the different stages of leaf senescence in Arabidopsis that are not specifically related to senescence associated gene expression.

## Materials and methods

### Comparative analyses of *ddc*, *ros1* and Col-0

The Arabidopsis hypermethylated *ros1* and hypomethylated *ddc* mutant lines, used previously by Chen *et al.* (2018), were grown in hydroponic culture under long days (16/8 h day/night) and compared with Col-0 wild type for visual symptoms of senescence. After seeding, the seeds were stratified at 4 °C for 5 d in the dark. ¼ Modified Hoagland was used, containing 1 mM NH_4_NO_3_, 1 mM CaCl_2_, 0.5 mM MgSO_4_, 1 mM KH_2_PO_4_, 1 mM KNO_3_, 100 µM NaFe(III)EDTA, 46 µM H_3_BO_3_, 9 µM MnSO_4_, 0.765 µM ZnSO_4_, 0.32 µM CuSO_4_, and 0.016 µM Na_2_MoO_3_. The air temperature was set at 20 °C during the day and 16 °C during the night. Photon intensity was 120–140 µmol m^−2^ and relative humidity was set to 55%. Sixteen 5-litre pots with five plants per pot were grown in a randomized complete block design. At least one plant per genotype was present in each pot. Flowering time was scored as the percentage of plants that showed visible transition of the shoot apical meristem to reproductive growth for each scoring date. Images of the plants were taken every 2–3 d and evaluated visually for symptoms of senescence. Experiments were conducted and repeated in two different laboratories (Hohenheim and Tübingen) with similar outcomes. First symptoms of senescence were scored as the date when visible yellowing appeared on the leaf rosette. Fifty percent senescence was scored as the date when approximately half of the leaf rosette showed visible discoloration. Statistical analysis was performed by *glimmix* and *lsmeans* in SAS. The three genotypes were then grown together for 50 d under short day conditions (8/16 h day/night), followed by a transition to long days. For Col-0, samples were taken at 30 d after sowing (DAS) (day 0 after light change), 44 DAS (day 14), 51 DAS (day 21), 60 DAS (day 30), and 90 DAS (day 60). The last samples were taken when senescence was complete for the whole plant. The above-ground biomass was separated into leaf, stem, and seed material and weighed separately. Statistical analysis was performed with R, interactions were analysed using the functions *aov*, *lm*, *lsmeans*, and *cld*, with *alpha*=0.05 and *adjust*=‘tukey’, and the *LSD.test* was performed following *aov*. The three genotypes were compared separately for rosette, stem and seed biomass.

### Leaf physiology and cytosine methylation

One hundred and twenty Col-0 plants were grown in soil under long day conditions. During vegetative growth, the leaves at positions 5–9 within the rosette were labelled from bottom to top for the following analyses: leaf 5, chlorophyll extraction; leaf 6, sugar content; leaf 7, whole genome bisulfite sequencing (WGBS); leaf 8, hormone analyses; and leaf 9, H_2_O_2_ content. Four developmental stages were chosen roughly corresponding to the following stages from the ‘timetable of Arabidopsis growth stages’ at https://www.arabidopsis.org/: 1: S5, inflorescence emergence (Bolting; Blt); 2: S6, flower production (Flowering; Flwr); 3: S6.3, 30% of flowers produced open (seed development; SD); and 4: S8, silique or fruit ripening (seed maturation; SM). Twenty plants were harvested at each developmental stage for further analyses.

### Leaf physiology

An automated colorimetric assay was performed and H_2_O_2_ levels were estimated as described by Bresson *et al.* (2018). Chlorophyll was extracted from 20 leaves per developmental stage. The fresh weight of each leaf was measured at harvest. The leaves were subsequently frozen in liquid nitrogen and then vacuum-dried. Once the dry weights had been measured, chlorophyll extraction in 80% acetone in phosphate buffer proceeded as described in Bresson *et al.* (2018).

Principal component analysis (PCA) was performed in R by using the *prcomp* function with *center=*T and *scale=*T and by using pixel counts from the automated colorimetric assay analysis for green, green-yellow, yellow, brown, and total leaf pixels and on leaf fresh weights. Linear regression analysis with k-mer validation was performed using the *ceret* R package. Data were indexed using the *createMultiFolds* function with *k=*5 and *times=*10. The method was adjusted using *trainControl*, with *method*=*‘repeatedcv’*, *number=*5, *repeat=*10, and *index*. The model was trained using *train*, with *method=‘lm’*. The chlorophyll concentration in dry weight was predicted with the following formula:


Chl/DW~percent green+percent green:g.norm+percent green:percent gy

where Chl/DW is mg chlorophyll per g dry weight, percent green is green pixel count/total pixel count, percent gy is green-yellow pixel count/total pixel count, and g.norm is normalization of green pixel count for each individual leaf towards the maximum green pixel count reached by each leaf position:


(Maximum green pixel countLeaf green pixel count)/Maximum green pixel count

Sugar content was analysed from leaf no. 6 in a Dionex/Thermo ICS 5000 system equipped with pulsed amperiometric detection. Hormone levels were quantified from leaf no. 8 by using GC-MS (Shimadzu TQ8040) in the splitless MRM mode.

### Nitrogen remobilization during senescence

The three genotypes were grown in 20 five-litre containers, as described for the previous experiment. The P concentration in the ¼ modified Hoagland used previously was noticed to have an inhibiting effect on plant growth. Here, KH_2_PO_4_ was reduced to 0.2 mM with the addition of 0.8 mM KCl to compensate for K nutrition. Plants were grown for 50 d under short day illumination, followed by a transition to long day illumination for flower induction. Samples were taken starting at 30 DAS (day 0) and continuing at 44 DAS (day 14), 51 DAS (day 21), 60 DAS (day 30), and 90 DAS (day 60). The last samples were taken when senescence was complete for the whole plant. The above-ground biomass was separated into leaf, stem, and seed and weighed separately. Statistical analysis was carried out with R, interactions were analysed using the functions *aov*, *lm*, *lsmeans*, and *cld*, with *alpha*=0.05 and *adjust*=tukey, and the *LSD.test* was performed following *aov*. The three genotypes for rosette, stem, and seed biomass were compared separately.

### qRT-PCR

We tested the gene expression of six genes that were selected for further analysis. RNA was extracted from the ground leaf powder remaining after the WGBS experiment (leaf no. 7) by using the Analytik Jena innuPREP Plant RNA Kit with guanidinium chloride. RNA quantity and quality were tested via NanoDrop 2000c and gel electrophoresis. cDNA was synthesized from 1 µg of the total extracted RNA by using the Quantitec Reverse Transcription Kit from Qiagen. qRT-PCR was performed with 15 ng cDNA per reaction in a Bio-Rad CFX 384 with the GreenMasterMix from Genaxxon Bioscience, according to the manufacturer’s protocol. PCR cycling parameters were set as follows: 95 °C for 3 min, 45 cycles of 3 s at 95 °C, 20 s at 60 °C, and a final melting curve of 65–95 °C with increments of 0.2 °C. The *AtACTIN2* gene was used as a reference gene for normalization (Supplementary Fig. S1). Six other genes were chosen for analysis: *AtROS1* (*AT2G36490*), *AtPHYC* (*AT5G35840*), *AT3G44530*, *AT1G44820*, *AT5G53120*, and *AT1G66890*. The primers used are listed in Supplementary Table S1. Because of the limited amount of sample material, qRT-PCR was performed from two biological replicates with three technical repetitions. The experiment was repeated until consistent results were achieved. Insufficient RNA was obtained in the extract from the last stage of the experiment, seed maturation, and hence the results presented here are from only one biological replicate. ΔΔ*C*_t_ values were calculated for each biological replicate. The time point ‘Flwr’ (flowering) was taken as the reference; therefore, its mean value always equals 1 after normalization.

For each gene and technical replicate:


$$\Delta C_{\text{t}}=C_{\text{tBlt}}\text{ mean}\left(C_{\text{tFLWR}}\right)$$

mean(*C*_tFlwr_) is the average *C*_t_ from all technical replicates

For each gene, technical replicate and time point:


$$\Delta \Delta C_{\text{t}}=C_{\text{tGene}}-\text{mean}\left(\Delta C_{\text{tactin}}\right)$$

mean(Δ*C*_tactin_) is the average Δ*C*_t_ from all technical replicates

ANOVA followed by Tukey’s honestly significant difference (HSD) test was performed in R to assess statistical significance as follows:


$$\text{aov}\left(2^{-}{}^{\Delta \Delta C_{\text{t}}}\sim \text{Bio}+\text{ Time Point}\right)$$

This function performs an ANOVA for the effects of Bio (biological replicate) and time point on the normalized ΔΔ*C*_t_ value.

HSD.test(model,‘Timepoint’) provides the letter description following the Tukey HSD test for statistical significance of time point on , while regulating for biological replicate.

### Hormones

Hormone levels were quantified from leaf no. 8. Five leaves were pooled at random to produce four replicates per developmental stage. After harvest, leaves were flash-frozen in liquid nitrogen and stored at −80 °C. The samples were ground twice for 0.5 min at 30 Hz with a Retsch Mixermill and were cooled with liquid nitrogen before and between milling steps. They were then extracted in 2 × 750 µl (total 1500 µl) ethyl acetate with 0.1% formic acid, containing the internal standard (3-hydroxybutyrate 60 ng, 9,10-dihydrojasmonic acid 80 ng, and 3-indole pyruvic acid 50 ng per ml), for 10 min in an ultrasonic bath. After centrifugation, the supernatant was used to quantify soluble hormones, whereas the pellet was hydrolysed to release any bound compounds. After removal of the solvent from the supernatant by using an Eppendorf vacuum concentrator (mode HV) at 30 mbar, 70 µl of a fresh 1:1 mix of methanol and trimethylsilyldiazomethane, Sigma-Aldrich) was added to the dry samples. GC-MS (Shimadzu TQ8040) in the splitless MRM mode was used for sample analyses. Hydrolysis of the dry pellet was performed using 200 µl of 3 M HCl and 200 µl of 3 M NH_3_.

### Hydrogen peroxide

H_2_O_2_ was quantified from 20 leaves per developmental stage exactly as described in Bresson *et al.* (2018). A stock solution was prepared containing 0.4 mg 5(6)-carboxy-2ʹ,7ʹ-dichlorofluorescin diacetate solved in 400 μl dimethyl sulfoxide, diluted 1:1 with distilled water. The working solution contained 400 μl of stock solution added to 39.6 ml MS medium. Individual leaves were weighed in and incubated in 1 ml working solution for 45 min at room temperature. The sample leaf was rinsed with distilled water and frozen in liquid nitrogen. An aliquot of 500 µl of 40 mM Tris buffer, pH 7, was added and the sample was homogenized. After centrifugation for 15 min at 14 000 *g* and 4 °C, the supernatant was measured on a plate reader (excitation: ~480 nm, emission: ~520 nm). On each sampling day, calibration was performed as follows. An aliquot of 0.755 ml of 0.5 M NaOH solution was mixed with 1 ml working solution. After incubation for 30 min at room temperature, 500 μl of 30% H_2_O_2_ solution was added to 156 μl deacetylated dye. The sample was shaken at room temperature for 1 h in the dark (holes were punched in the tube cap to allow gas escape). After the addition of 226 μl Tris buffer, the fully oxidized deacetylated dye was measured photometrically at 520 nm.

### Whole genome bisulfite sequencing and analysis

The quality of the raw reads was assessed using FastQC Version 0.11.9. Trimmomatic 0.39 was used for the trimming and filtering of the raw reads at the following settings: SLIDINGWINDOW:4:15, LEADING:3, TRAILING:3, ILLUMINACLIP:2:30:10:1, MINLEN:36, HEADCROP:15. Sequence alignment was performed using Bismark 0.22.3 at default settings (Krueger and Andrews, 2011). The reference genome was TAIR10 assembly. Cytosine methylation was called with the bismark_methylation_extractor at the following settings in the command line: -p --comprehensive --bedGraph --CX --multicore 3 --cytosine_report. After analysis of coverage distribution, cytosines with a coverage of <3 or >40 were removed from further analyses by using R. Statistical analysis of differential methylation was carried out using the DSS package from bioconductor (Park and Wu, 2016). All 12 samples were combined using the *makeBSseqData* function. Data transformation and analysis were performed with *DMLtest*. For general descriptive analyses, the mean value for each position was taken from the resulting tables following all possible pairwise comparisons of the four time points under investigation. Differential methylated loci (DMLs; 25% change) were called using the *callDML* function with *p.threshold*=0.05. Differential methylated regions were called using the *callDMR* function with *p.threshold*=0.05.

PCA and cluster analysis were carried out on the weighted means for the methylation of 50 bp bins of the entire genome and of all single cytosines recognized as DMLs in any of the possible pairwise comparisons. The R function *prcomp(center=T)* was used for PCA analysis followed by *ggbiplot* for plotting. Cluster analysis was performed with the R function *dist(method=‘manhattan’)* followed by *hclust(method=‘ward.D’)* and plotted via *plot()*.

The locations of transposable elements (TEs) and gene open reading frames (ORFs) were downloaded from the TAIR10 assembly and customized into *bed* file format by using python. The region lying 2500 bp upstream of the ATG start codon of every ORF was also recorded in *bed* format and analysed as the 2500-bp promoter. The *bedtools* functions *intersect* and *closest* were used to find the locations of all DMLs and differentially methylated regions (DMRs) with respect to the above-mentioned genomic elements.

The locations of all W-boxes (*TTGACT/C*) were sought in the TAIR10 assembly with the regular expression function of python, namely *re.finditer*. The start and end position, together with the chromosome number, was recorded in a *bed* file format and further processed with bedtools *intersect* and *closest*. Bootstrap analysis was performed to estimate the statistical significance of the cytosine methylation in W-boxes compared with their genomic context. The average cytosine methylation level for all CHH and CHG in ORFs, TEs and 2500 bp promoters was calculated separately. These were then compared with the sub-samples comprising all CHH and CHGs present in W-boxes. 10 000 random samples were drawn without replacement, with the same size as the sub-samples, and the percentage of them with the same or more extreme average was taken as the *P-*value estimate.

DMRs in the *ddc* mutant were quantified from the data of Stroud *et al.* (2013) according to Jühling *et al.* (2016). Metilene 0.23 was used at the default setting and the minimum distance between the adjacent cytosines was set at 100 bp. DMRs were intersected with the locations of known genomic regions in the face of TEs, ORFs and the −2500 bp regions from the ATG start codon of ORFs (2500 bp promoters) via the bedtools command *intersect*. The Softberry Nsite-PL (Solovyev *et al.*, 2010; Shahmuradov and Solovyev, 2015) algorithm was employed to locate known transcription factor binding motifs within DMRs intersecting with 2500 bp promoters. All transcription factor binding motifs specific for Arabidopsis were then used for motif pattern recognition via MEME (Bailey *et al.*, 2009).

### Protein expression

WRKY18, WRKY25, and WRKY53 were expressed in *Escherichia coli* with an N-terminal-fused 6×His-tag, as described by Doll *et al.* (2020). The *E. coli* were grown overnight in 5 ml selective medium (5 ml LB + 5 µl ampicillin 100 µg ml^−1^). An aliquot of 50 ml of the same selective medium was then inoculated with the pre-culture and shaken at 200 rpm and 37 °C for 1.5 h. At an OD_600_>0.6, the culture was induced with 50 µl 1 M isopropyl β-D-1-thiogalactopyranoside and incubated overnight (18 °C, 180 rpm). Cells were collected (30 min centrifugation at 1700 *g*, 4 °C) and suspended in protein extraction buffer (200 ml containing 10 mM HEPES, 50 ml 1 M KCl, 40.8 ml of 98% glycerol). After sonication, the solution was centrifuged at 4 °C and 12 000 *g*. The Bradford assay was used to detect protein concentrations in the crude extract.

### Verification via western blot

The presence of 6×His-tagged protein in the crude extract was validated via western blot analysis. For SDS-PAGE, 12.8 ml water, 10.7 ml of 30% acrylamide, 8 ml of 1.5 M Tris pH 8.8 and 320 µl of 10% SDS were added together. An aliquot of 320 µl of 10% ammonium persulfate was mixed briefly with 32 µl tetramethylethylenediamine. The gel was then poured into frames that were subsequently sealed with 1% agarose solution. After the acrylamide had polymerized, the gel was packed and left at 4 °C until needed. SDS-PAGE separation was performed with 25 µg raw protein extract for approximately 2 h at 330 A. Semi-dry transfer to a binding membrane was performed using a transfer buffer (192 mM glycine, 25 mM Tris, 200 ml of 100% ethanol) and incubation at 300 mA for 1 h.

Blocking solutions were prepared comprising 3% and 1.5% solutions of milk powder in Tris-buffered saline–Tween 20 (TBS-T) buffer. The membrane was first incubated in the 1.5% solution at 4 °C for 30 min and then blocked with 3% milk powder in TBS-T for 1 h. After three washing steps in TBS-T buffer for 10 min each, the membrane was covered in a 1:5000 dilution of antibodies (anti-His–horseradish peroxidase (HRP) conjugated antibodies, Qiagen) and incubated for 60 min with slow rotation, followed by three more rounds of washing, and finally, detection.

### DNA–protein interaction enzyme-linked immunosorbent assay

DNA–protein interaction enzyme-linked immunosorbent assay (DPI-ELISA) was performed following the instructions of Brand *et al.* (2010). A 29-bp long biotinylated artificial sequence, containing three W-boxes, was used for the assay. The forward sequence was:

GGAACTTGACCATTTGACCTTGACCATGG (Art. 3*Wbox F—Biotin) and the reverse sequence was

CCATGGTCAAGGTCAAATGGTCAAGTTCC (Art. 3*Wbox R). The sense and antisense oligonucleotides were diluted (2 pmol µl^−1^), heated for 5–10 min at 95 °C and left to cool. Streptavidin-coated pre-blocked clear 96-well plates (Nunc Immobilizer Streptavidin, Thermo Fisher Scientific) were used for the assay. The wells were pre-washed 3 × 200 μl per well with 5×SSCT buffer (750 mM NaCl, 75 mM sodium citrate, 0.05% (v/v) Tween-20). Two concentrations of primers were tested: 0.2 and 0.02, corresponding to 20 and 2 pmol 60 µl^−1^. The annealed primer pairs were diluted to their final concentration in 5×SSCT and added to the streptavidin-coated plate, which was then incubated at room temperature for 1 h (60 µl per well). The wells were washed in 2 × 100 μl 2×SSCT (300 mM NaCl, 30 mM sodium citrate, 0.05% (v/v) Tween-20) and 2 × 100 μl protein dilution buffer. Up to five crude protein extract concentrations were tested on the immobilized ds-biotinylated DNA (0.5 μg, 5 μg, 25 μg, 50 μg, 100 μg in 60 μl per well). After being incubated for 1 h at room temperature, the plates were washed three times in 100 μl TBS-T. α-His–HRP antibody conjugate (Qiagen) diluted 1:1000 in TBS-T was added to the wells and incubated for 1 h at room temperature (60 μl per well) with mild agitation. After being washed in 2 × 100 μl TBS-T and 2 × 100 μl TBS, the plates were prepared for photometric detection by being incubated with OPD solution (60 μl per well comprising 4 mg *ortho*-phenylenediamine (OPD tablets from Sigma), 3 μl 30% H_2_O_2_ in 6 ml CP-buffer (10 mM Na_2_HPO_4_, 100 mM citric acid, pH 5 with NaOH)) in the dark for a maximum of 30 min. After the addition of an equal volume of stopping solution (60 μl 2 M HCl), the plate was kept for a further 10 min in the dark. Absorbance was measured at 492 nm by using 650 nm (plate background) as a reference wavelength in an ELISA reader.

### Cytokinins

For the estimation of cytokinin levels in the early stages of leaf senescence, Col-0 plants were grown in soil under short days for 20 d, after which flowering was induced via transition to long days. A first sample was taken at 20 DAS, as an estimation for cytokinin levels before flower induction (Pre-F). Second and third samples corresponded to the bolting (Blt) and flowering (Flwr) stages of the previous experiment. Leaf no. 8 was harvested from 12 plants per sampling point. The leaves were pooled into three biological replicates. The levels of *trans*-zeatin, *trans*-zeatinriboside, *cis*-zeatinriboside and kinetin were estimated as follows. As an internal standard, 20 ng [^2^H_5_]-*trans*-zeatin was added to the sample and incubated for 15 min. Extraction was performed with 1.5 ml of 0.1% formic acid and 600 mg ceramic beads in a FastPrep (6.5 m s^−1^ for 45 min) followed by incubation in a rotating vortex (99 rpm and shaking) at room temperature for 30 min. Samples were centrifuged at 12°C at 16 000 *g* for 30 min. The supernatant was collected and extraction was repeated with 1.2 ml formic acid (0.1%). SPE columns (Waters Oasis MCX, 150 mg) were conditioned with 5 ml methanol followed by 5 ml of 0.5 M formic acid before the sample was applied. Samples were washed first with 3 ml of 0.5 M formic acid and then with 0.1% formic acid–methanol (95%/5% v/v). Cytokines were eluted with 3 ml NH_4_OH–methanol (1%/60% v/v) and elutions were dried under a nitrogen flow in an ExcelVap at 22 °C. Pellets were resuspended in 150 µl of 0.1% formic acid–methanol (95%/5% v/v). Aliquots of 15 µl were analysed by LC-MS.

## Results

### Vegetative and reproductive growth, leaf senescence, and nitrogen remobilization in *ddc* and *ros1*

**Fig. 1.**
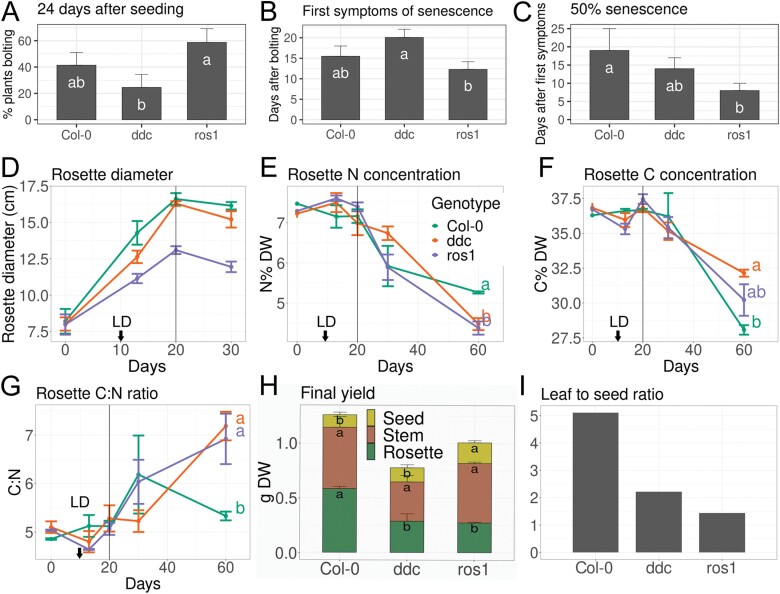
Flowering time and senescence of Col-0, *ddc*, and *ros1*. (A) Percentage of plants that had undergone the transition of the shoot apical meristem (SAM) to flowering at 24 d after sowing in soil. (B) First symptoms of senescence of the whole rosette after appearance of the first bolt. (C) Days until 50% senescence (as visual count of the total rosette) was reached, since first symptoms were observed. (D–G) Time course for Col-0, *ddc*, and *ros1* in hydroponics (sampling started at day 30 after seeding, this time point was set to 0 in the graph) with flowering induced via transition to long day (LD) regime at 10 d after first sampling (indicated by black arrow). All plants exhibited visual transition to reproductive growth in the SAM at 20 d after first sampling (grey line). (D) Rosette fresh weight; (E) nitrogen concentration in the rosette; (F) carbon concentration in the rosette; (G) carbon to nitrogen ratio of the rosette; (H) rosette, stem and seed final dry matter yield. Statistical comparison was made separately for rosette, stem, and seed biomass. Significant differences are indicated by different letters, where *a* is significantly larger than *b* with *P*<0.05. (I) Leaf to seed ratio. Error bars indicate standard error; (A–C) were estimated from five to six plants per genotype; (D–I) were estimated from four plants per genotype; letters indicate significant differences estimated by LSD test, following ANOVA at *P*<0.05.

The involvement of cytosine methylation in flowering time and leaf senescence was first investigated in the hypermethylated *ros1* (Gong *et al.*, 2002) and the hypomethylated *drm1*,*drm2*,*cmt3* (*ddc*; Cao and Jacobsen, 2002) mutants. These mutants were grown in soil under long day conditions. Plants were scored for the transition to reproductive growth, for the first visible symptoms of senescence, and for 50% senescence of the leaf rosette (Fig. 1A–C). Both mutants showed compromised rosette growth and smaller rosette diameter compared with Col-0, with the mutant *ros1* having the more severe phenotype (Supplementary Fig. S2). Aberrant methylation in these mutants tended to alter the flowering time and senescence progression, with the *ddc* mutant flowering slightly later and having delayed first symptoms of senescence. In these conditions, the *ros1* mutant tended to flower approximately 10 d earlier and exhibited earlier senescence compared with Col-0, although these differences in flowering time were only significant between the two mutants, but not between mutants and the wild type (Fig. 1A, B). Once the first symptoms of senescence had appeared, both mutants executed the senescence programme faster than Col-0, with *ros1* being the more rapid (Fig. 1C). Whereas *ddc* had a similar total lifespan to that of Col-0, *ros1* had an overall shorter lifetime.

To examine possible physiological reasons and consequences of the aberrant senescence phenotype, carbon (C) and nitrogen (N) remobilization during senescence were studied in these mutants in hydroponic culture. The plants were grown under short days, followed by a transition to long days to synchronize flower induction. The first samples were taken at 30 d after sowing. The three genotypes behaved as in the previous experiment, with Col-0 establishing the largest rosette biomass and *ros1* the least (Fig. 1D; Supplementary Fig. S2). In agreement with previous reports (Diaz *et al.*, 2008), the N concentration in the rosette declined with proceeding senescence, but the initially delayed beginning of senescence of *ddc* was correlated with a slightly later initiation of bulk N remobilization than in the other lines (Fig. 1E). Interestingly, *ros1* and *ddc* remobilized approximately 40% more N than Col-0 out of the rosette until the end of the senescence process, when the leaves from the rosette had died (Fig. 1E). In contrast, the total C content of their leaves at the last harvest date was higher than that in Col-0 (Fig. 1F), corresponding to a higher overall leaf C:N ratio at the end of senescence (Fig. 1G). Final seed yield was higher in *ros1*, and *ddc* and *ros1* had less dry rosette biomass (Fig. 1H). This resulted in a lower overall leaf to seed ratio in senescent mutants, compared with Col-0 (Fig. 1I), suggesting that nutrient (nitrogen) remobilization and progression of senescence in *ddc* and *ros1* were affected, in agreement with the previous experiment.

### Flowering, leaf senescence, and N remobilization with transient N shortage in *ddc* and *ros1*

**Fig. 2.**
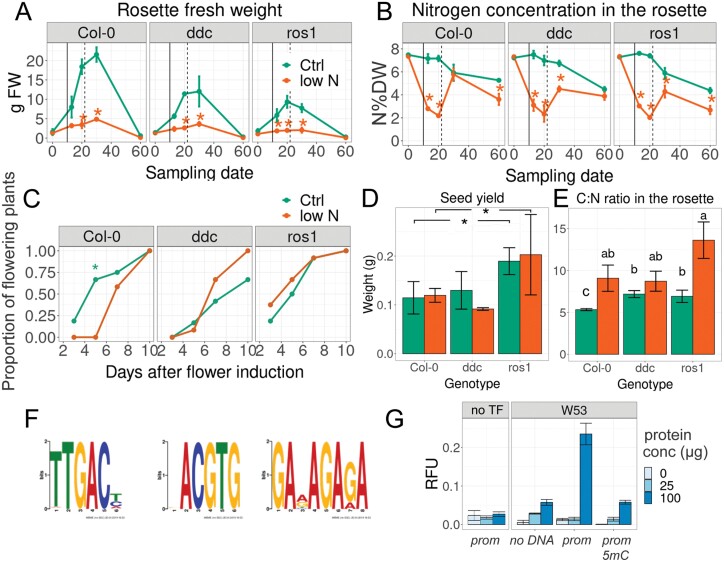
Temporary nitrogen withdrawal for 3 weeks in Col-0, *ddc*, and *ros1*, and cytosine methylation targets in *ddc* and effects. Green, full nutrition; orange, withdrawal of N starting at day 0 (30-day-old plants). On day 10, the light regime was changed; on day 21, the plants were resupplied with nitrogen. (A) Rosette fresh weights. (B) Nitrogen concentration in the rosette dry weight (N%DW). (C) Percentage of plants that visibly showed a bolt. The solid vertical line indicates induction of flowering via change in light regime and the dashed vertical line indicates nitrogen resupply. Significant differences are indicated by stars, *P*<0.05. Comparisons were made between the two treatments, separately for each genotype and sampling date. (D) Seed biomass. (E) carbon to nitrogen ratios in the rosette. Letters indicate significant differences as indicated by lsmeans test adjusted for Tukey HSD, following ANOVA at *P*<0.05. Stars indicate significant effect of genotype only, without interactions between treatment and genotype (*n*=4; means ±SEM). (F) Top three most common transcription factor binding motifs found in DMRs between *ddc* and Col-0 intersecting with 2500 bp promoters. E-values were: 1.5e-006, 56, and 2.5, respectively. (G) Cytosine methylation influence on the binding of WRKY53 by DPI-ELISA. Colour codes indicate the concentrations of protein (in µg per 60 µl reaction). prom: non-methylated, artificial promoter (concentration: 0.33 pmol µl^−1^); prom 5mC: methylated, artificial promoter (*n*=2; means ±SEM).

As prolonged N deficiency was associated with massive overall decreases in cytosine methylation in maize roots (Mager and Ludewig, 2018), and maintained changes in the cytosine methylation patterns in the next generation were observed with differential N supply in rice (Kou *et al.*, 2011), we tested the impact of temporary N withdrawal on the flowering time and senescence of the three genotypes grown in hydroponics. Nitrogen was reduced to 5% of the initial concentration (to 50 µM) for 3 weeks and was then resupplied. Flowering was induced by a change in the light regime (to long days) at 10 d after N withdrawal. All three genotypes quickly reduced their growth rates, resulting in a reduced rosette biomass, because of low N (Fig. 2A). Col-0 produced significantly more rosette biomass under control conditions (Fig. 2A), whereas *ros1* produced the least biomass, especially when N was withdrawn. However, in contrast to Col-0, neither *ddc* nor *ros1* recovered the N concentration in the rosette directly following resupply (Fig. 2B). Nitrogen withdrawal delayed the flowering time in Col-0 but not in the two mutants, and *ddc* showed a tendency to flower slightly earlier as a result of the treatment (Fig. 2C). Furthermore, *ros1* showed the highest seed yield, even after transient N deficiency (Fig. 2D). Again, *ros1* had the lowest N and highest C concentrations in the rosette at the final time point, corresponding to a higher C:N ratio (Fig. 2E).

### Cytosine methylation in WRKY binding sites is functionally important and affected in *ddc*

We considered the possibility that the aberrant remobilization and senescence phenotype in *ddc* was caused by an interference of cytosine methylation in transcription factor binding sites near genes. To test this hypothesis, we analysed the methylome datasets of *ddc* and Col-0 provided by Stroud *et al.* (2013). *ddc* had 18% less CG methylation and 89% and 56% reduced methylation in the CHG context (in which H means any base out of A,T,C) and in the CHH context (Supplementary Fig. S3A), respectively. This led to 228 DMRs of which 186 intersected with annotated genomic elements, the majority being found in TEs or −2500 bp from the TSS of gene ORFs (Supplementary Fig. S3B). One hundred and seventy-eight motifs were recognized within DMRs that intersected with 2500 bp promoter regions; most of these, however, appeared only once. The only significant repetitive occurrence was that of the *TTGAC(T/C/A*), corresponding to the conserved W-box element recognized by WRKY TFs (Fig. 2F), which appeared 17 times across 12 DMRs. Eight of the 12 DMRs, containing 13 of the 17 binding sites, were located within TEs in close proximity to genes. The second most differentially targeted motif was part of a core motif recognized by bZIP transcription factors (*ACGTG*), which appeared seven times in seven DMRs. Five out of these seven occurrences were within TEs in close proximity to genes. These results hint at the possibility that the loss of methylation in CHG and CHH contexts affected the WRKY and/or bZIP transcription factor network, with TEs in relatively close proximity to gene TSS, playing a role in their regulation.

Using artificial binding targets, we verified that methylation massively inhibited *in vitro* DNA binding of key transcription factors of senescence, namely WRKY53 (Fig. 2G), WRKY18, and WRKY25 (Supplementary Fig. S4), by biochemical binding assays (Brand *et al.*, 2010). Whereas WRKY18 bound the most strongly to the artificial 3× W-box target when non-methylated (Supplementary Fig. S4A, C), the interaction of all three proteins with the artificial promoter sequence was greatly impaired by (complete) cytosine methylation of the artificial target (Fig. 2G; Supplementary Fig. S4).

### Relationship between reproductive stage, rosette size, leaf coloration, chlorophyll, and leaf physiology in Col-0

**Fig. 3.**
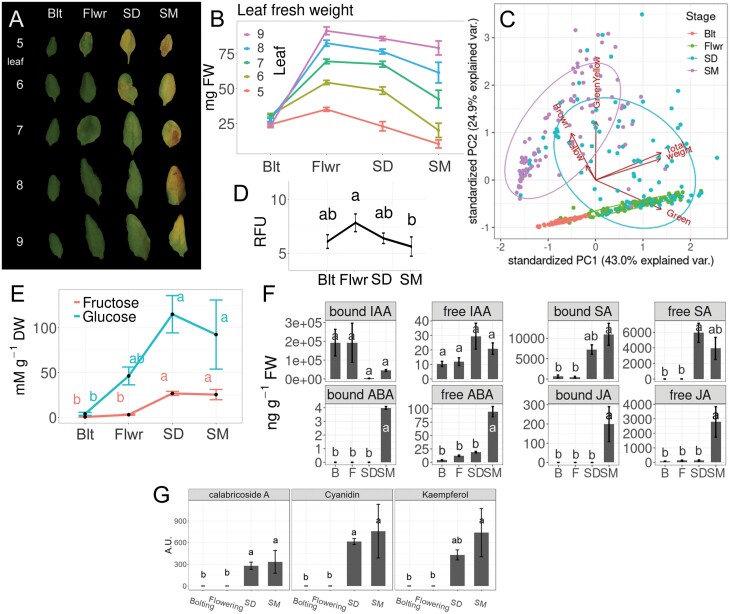
Progression of senescence in leaf nos 5–9. (A) Fresh weight measured on per leaf basis (*n*=20; means ±SEM). (B) PCA on pixel counts for green, green-yellow, yellow, and brown coloration, total pixel count, and leaf fresh weight distinguishing the four developmental stages: bolting (Blt/B), flowering (Flwr/F), seed development (SD) and seed maturation (SM). (C) Representative leaves at the four stages. (D) Hydrogen peroxide in leaf no. 9. RFU, relative fluorescence units. (n=20; means ±SEM; LSD test following ANOVA.) (E) Glucose and fructose concentrations in leaf no. 6 (*n*=4; means ±SEM; LSD test following ANOVA). (F) Concentration of hormones, precursors, and defence-related compounds: auxin (IAA, 3-IPA), abscisic acid (ABA), salicylic acid (SA), jasmonic acid (JA), and camalexin in soluble (free) and hydrolysable (bound) form (*n*=3; means ±SEM; LSD test following ANOVA, where *a* is significantly larger than *b* with *P*<0.05).

In order to analyse genome methylation changes within individual leaves along the senescence process, we chose four physiologically relevant time points in the reproductive development of the plant: bolting (Blt), flowering (Flwr), seed development (SD), and seed maturation (SM) (Supplementary Fig. S5). Leaves were marked with coloured threads shortly after their appearance so that, even at later stages, the leaves were distinguishable and could be numbered according to their age (with no. 1 being the oldest and first emerging true leaf). Leaf nos 5–9 exhibited sequential senescence, with leaf no. 5 showing the earliest symptoms attributable to chlorophyll degradation, namely as early as the initiation of flowering (Fig. 3A; Supplementary Fig. S6). All leaves enlarged and gained weight until Flwr, the second time point (Fig. 3B). An automated colorimetric assay (Bresson *et al.*, 2018) trained on leaf no. 5 precisely predicted chlorophyll concentrations (adjusted *R*^2^=0.89) in leaf nos 6–9. The colorimetric and visible loss of green coloration and its variation (Fig. 3A; Supplementary Fig. S6) were largest at the third time point (SD), as indicated by PCA (Fig. 3C; Supplementary Fig. S6). By normalizing leaf weight to a scale between −1 and 1, different leaves of distinct individual plants were successfully assigned directly to certain stages, despite their slightly disparate size and time after seeding due to biological variation (Supplementary Fig. S6). A peak of H_2_O_2_ was observed at the flowering stage, when leaf nos 6 and 9 approached their maximum size and weight (Fig. 3D; Supplementary Fig. S6). Glucose increased as early as Flwr, when the first reduction in chlorophyll occurred (Fig. 3E; Supplementary Fig. S6), followed by an increase of fructose at SD, when glucose concentrations peaked (Fig. 3E).

Leaf no. 8 was then analysed for various phytohormones, namely the auxin indole acetic acid (IAA), its precursor indole-3-pyruvate (3-IPA), abscisic acid (ABA), salicylic acid (SA), jasmonic acid (JA), and defence-related anthocyanins and camalexin. Whereas the phytohormones gibberellic acid, auxin and cytokinin are considered to inhibit senescence, ABA, JA, and SA are inducers of senescence (Schippers *et al.*, 2007; Jibran *et al.*, 2013). Hydrolysable (bound) IAA tended to decrease between Flwr and SD, while free IAA tended to increase at this time point (Fig. 3F) and its precursor 3-IPA was only elevated at SM (Supplementary Fig. S7). Free SA also increased between Flwr and SD (Fig. 3F). This occurred after the intracellular H_2_O_2_ had reached its peak (Fig. 3D) and was paralleled by an increased amount of the anthocyanins calabricoside A, cyanidin, and kaempferol (Fig. 3G), as well as the defence-related phytoalexin camalexin in its free form (Supplementary Fig. S7). This indicates that the progression of senescence was associated with the activation of defence mechanisms of the plant between Flwr and SD, before JA and ABA increased. These two phytohormones increased later at SM, both in their bound and active free forms (Fig. 3F).

### Time course of cytosine methylation changes in leaf 7 during ageing and senescence

Because of the phenotypes of the two methylation mutants (Figs 1, 2) and the inconsistent methylome changes reported previously to be associated with leaf senescence (Trejo-Arellano *et al.*, 2020; Yuan *et al.*, 2020), we decided to analyse whole genome cytosine methylation at the four stages (Blt, Flwr, SD, and SM) in leaf no. 7. High conversion efficiency and the sequencing measures indicated the high overall quality of our data (Supplementary Table S2). Use of triplicates ensured sufficient statistical power for the subsequent analyses and allowed an estimate to be made of the rate of stochastic variation in the progression of leaf decay. A total of 455 047 DMLs (25% change) were identified in the chromosomal DNA, with approximately 78% being unique between the six pairwise comparisons of stages. Most DMLs were found in the CHH context (169 015), followed closely by the CG context (163 909), with the least DMLs in the CHG context (122 123).

**Fig. 4.**
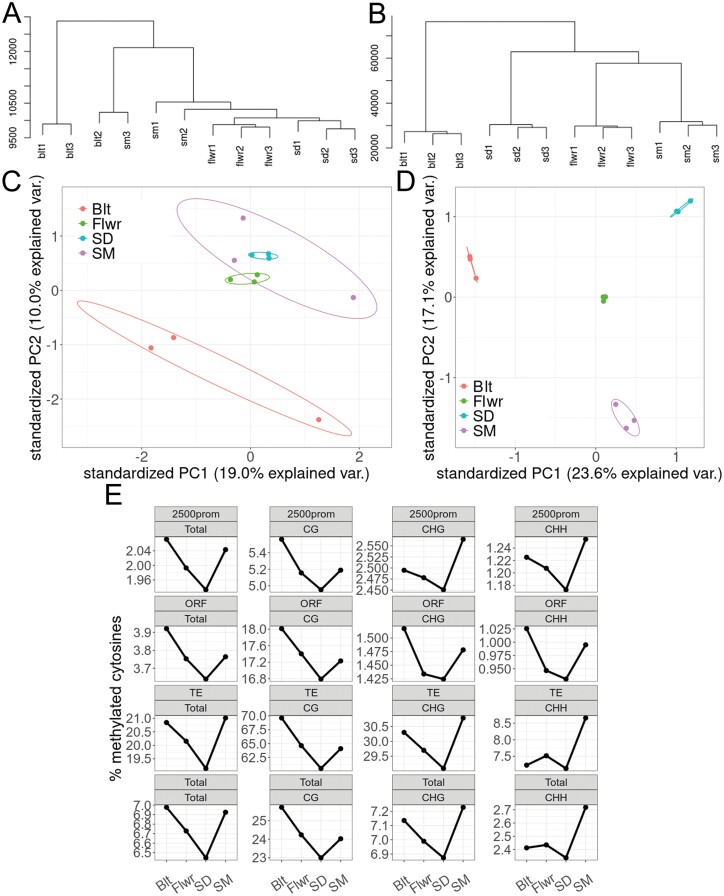
Cytosine methylation in leaf no. 7. (A–D) Reproducibility of the WGBS experiment. (A) Cluster dendrogram of the mean methylation calculated at 50 bp bins. (B) Cluster dendrogram of all cytosines categorized as DMLs in at least one pairwise comparison. (C) PCA analysis of the mean methylation calculated at 50 bp bins. (D) PCA analysis of all cytosines categorized as DMLs in at least one pairwise comparison. (E) Methylation of cytosines in 2500 bp promoter regions, open reading frames (ORF), transposon elements (TE), and total methylation of the whole genome (Total) during four developmental stages, analysed independently of context and separately in all three cytosine contexts (CG, CHG, and CHH).

To estimate the random noise and stochastic variation of cytosine methylation and the ability of the statistical package to pick stage-defining DMLs, we performed clustering and PCA on the methylome data with 50 bp windows with weighted means (Fig. 4). We first asked whether each methylome dataset was strictly associated with the chosen individual developmental stage, i.e. whether triplicate datasets from each developmental stage clustered together, but separately from the others. This analysis revealed two outliers of the complete methylome datasets at Blt and SM, which had less technical sequencing quality than the other samples (Supplementary Table S2). These were grouped together in the cluster analysis and separated the samples in PCA component 1 (Fig. 4A, C). DMLs of the four stages, however, were well-defined on both graphs, allowing us to select against random noise to pick stage-defining DMLs (Fig. 4B, D). DMLs appeared throughout the chromosome but the densest DML regions with higher average differential methylation were found around the centromere. In this region the highest concentration of TEs was found (Supplementary Fig. S8). Furthermore, the average methylation levels of all cytosines along different genomic features were estimated (Fig. 4E). There was progressively more hypo- than hypermethylation between Flwr and SD, but this reversed with the maturation of the seeds (SM) (Fig. 4E; Supplementary Fig. S7B). Most differences occurred, as expected, around the centromers, where methylation levels were intrinsically higher than in other genomic regions (Supplementary Fig. S7B). The total average cytosine methylation changes were small overall, but methylation dropped initially in all contexts examined and reached a minimum at SD (Fig. 4E). This drop was strongest in the CG context. The last stage of chlorophyll degradation and the beginning of desiccation during SM corresponded to a period of moderate *de novo* methylation that recovered to total pre-flowering methylation levels. Interestingly, however, the CG context did not recover completely, whereas cytosines in the CHG and CHH contexts in TEs and 2500 bp promoters reached levels higher than those beforehand (Fig. 4E). The largest reductions in methylation were observed in the CG context in TEs. Inhibition of the maintenance methylation during cell division and active RdDM might have been responsible for this finding and are in agreement with such patterns.

**Fig. 5.**
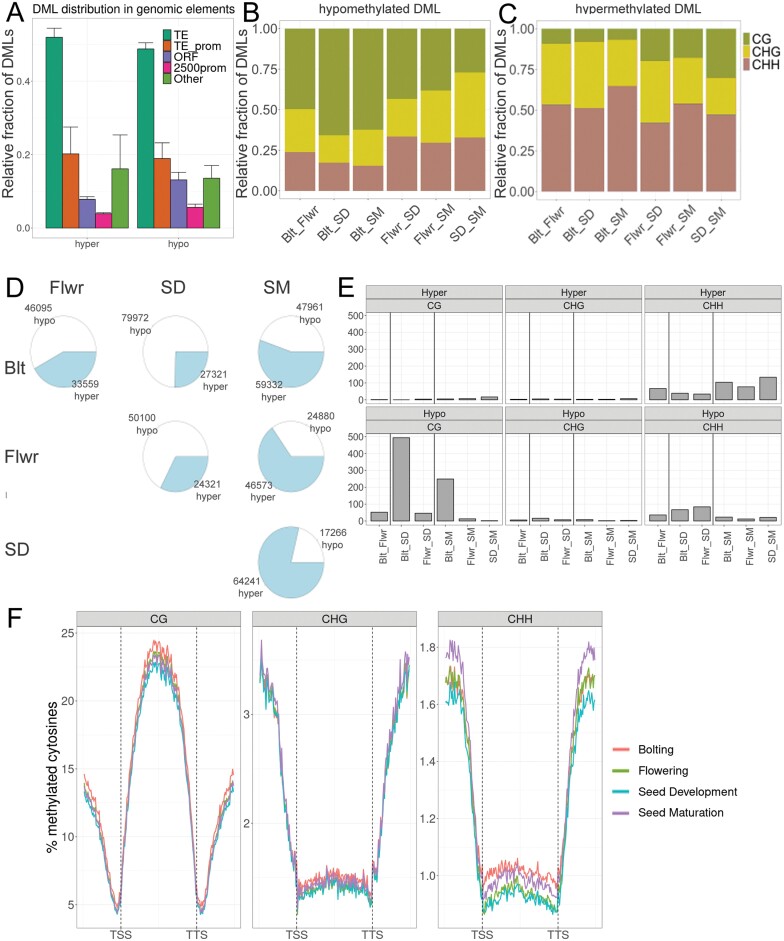
Relative distribution of differentially methylated loci (DML) in genomic contexts, hypo- and hypermethylated loci, and differentially methylated regions (DMRs). (A) Proportion of DMLs in TEs outside of promoters (dark green), TEs within 2500 bp promoters (orange), ORFs (violet), 2500 bp promoters that lack TEs (magenta) and intergenic regions (light green). Error bars indicate SEM. (B, C) Proportion of hypomethylated (B) and hypermethylated (C) DMLs in CG (green), CHG (orange), and CHH (violet) contexts. (D) Pairwise comparison of hypo- (white) and hypermethylated (light blue) loci in all pairwise comparisons. (E) DMRs in the six pairwise comparisons separated by cytosine context. (F) Average methylation levels of genes longer than 400 bp in three cytosine contexts. Regions 1000 bp upstream of transcription start sites (TSS) and 1000 bp downstream of transcription termination sites (TTS) were analysed together. Note the different *y*-axis scale for each context.

Differential methylation occurred mostly in TEs, followed by ORFs, and was found to be lowest in 2500 bp promoters (Fig. 5A). Interestingly, a large proportion of TEs containing DMLs intersected with ORFs and their 2500 bp promoters (Fig. 5A). About half of the loci that were hypomethylated between Blt and Flwr were in the CG context and one-quarter were in CHG and CHH contexts, respectively. About two-thirds of all hypomethylated loci were in the CG context (Fig. 5B). In contrast, hypermethylation predominantly occurred in CHG and CHH contexts throughout the observed stages (Fig. 5C). Stage-by-stage comparisons revealed gradual changes between stages and that hypomethylation dominated between Blt, Flwr, and SD, whereas hypermethylation dominated between SD and SM, with about three-quarters of DMLs being hypermethylated (Fig. 5D). The overall pattern of methylation along genes and their neighbouring 1000 bp regions was almost unaffected in the CG and CHG cytosine contexts, but mild differences were observed in the CHH context (Fig. 5F). In all contexts, the methylation around the TSS and TTS sites was particularly low and minor changes occurred at these sites. In the CHH context, methylation in genes was elevated at Blt and SM, while upstream promoter and downstream regions were only higher in SM, relative to Flwr and SD (Fig. 5F). This corroborates the differential changes in the CHH context described above at late senescence stage.

### Distribution and characteristics of differentially methylated regions

We found a relatively small number of DMRs associated with the different leaf stages, which precisely followed the patterns of DMLs (Figs 4E, 5E). Most DMRs (494) were observed in the CG context between Blt and SD, followed by Blt and SM (251). We noted that these numbers were not the sum of DMRs found between individual time point comparisons (each <50), meaning that DMRs developed gradually with time as a superposition of contexts (Fig. 5E). DMRs in CHH (total number: 683) were primarily hypomethylated until SD, whereas hypermethylated CHH DMRs were present throughout the experiment. DMRs in the CHG context were negligible throughout the process (Fig. 5E).

In order to recognize genes that were potentially influenced by DMRs with regard to their gene expression, we selected all ORFs and 2500 bp upstream regions containing DMRs. One hundred and thirty-two genes had at least one DMR intersecting with their ORF (Supplementary Table S3) but no significant enrichment was found when we probed the geneontology.org database or senescence-associated genes (Breeze *et al.*, 2011). Some notable groups included 10 genes involved in lipid metabolic processes, 12 genes involved in the hormonal response, 25 genes involved in stress responses, 16 genes involved in phosphorus metabolism, and 13 genes involved in protein phosphorylation. Interestingly, three WRKY transcription factor genes were found with DMRs located in their ORF, namely *WRKY18*, *WRKY26*, and *WRKY50*. Moreover, *CYTOKININ OXIGENASE/DEHYDROGENASE 1* (*CKX1*), which codes for the protein involved in catalysing the degradation of cytokinins, was in this group. A further 581 genes were found with at least one DMR intersecting with the region 2500 bp upstream of their TSS (Supplementary Table S4). Again, no significant enrichment was found when we probed databases, such as geneontology.org. Of these genes, 25 were involved in reproduction, 11 in the post-transcriptional regulation of gene expression, 42 in cellular component organization or biogenesis, 21 in peptide metabolic processes, and 10 in methylation. Genes worth mentioning with DMRs in the promoter were *ROS1*, *SUVH1*, which is involved in methylation recognition, and the phytochrome photoreceptor gene *PHYC*. The glutamine synthetase gene *GLN2*, three WRKY transcription factor genes (*WRKY41*, *WRKY48*, and *WRKY64*), two bZIP transcription factor genes (*bZIP23* and *bZIP60*), five NAC transcription factor genes (*NAC001*, *NAC003*, *NAC019*, *NAC071*, and *NAC100*) and one MYB gene (*MYB62*) also had DMRs in their promoters. None of these genes is, however, considered a central regulator of senescence, of flowering, or of pathogen defence, with the exception of *NAC019*, which is potentially involved in senescence.

### Cytosine methylation and gene expression

**Fig. 6.**
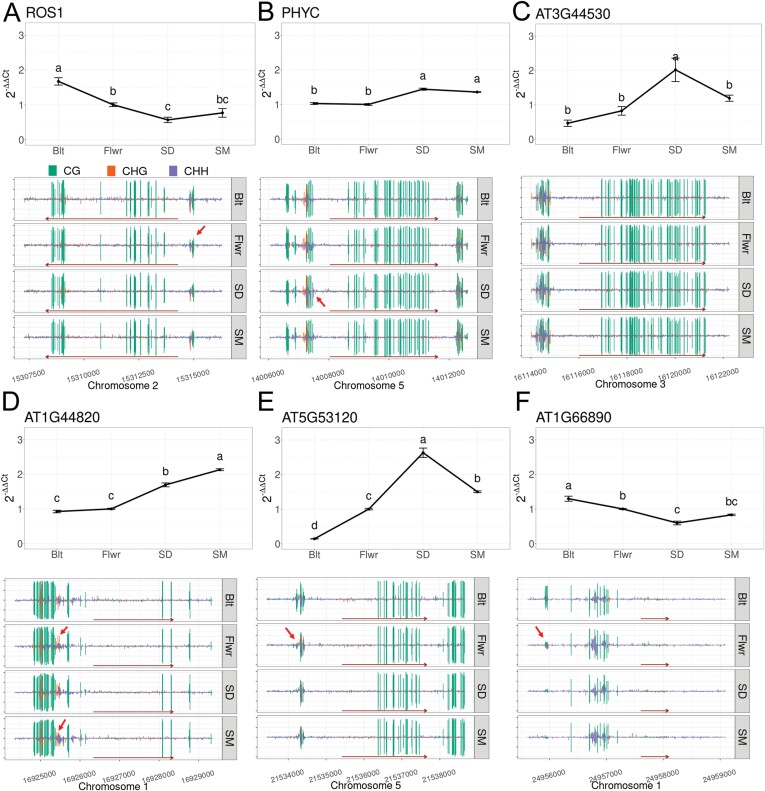
Patterns of gene expression (qRT-PCR) and cytosine methylation (CG, green; CHG, orange; CHH, violet) in representative genes. *ROS1* (A), *PHYC* (B), *AT3G44530* (C) (no DMR in this gene and promoter), *AT1G44820* (D), *AT5G53120* (E), and *AT1G66890* (F) for time points Blt, Flwr, SD, and SM. Dark red arrows along the *x*-axis of the gene methylation graphs indicate the gene span and direction. The small red arrows point at differentially methylated regions. For gene expression analysis, two pooled biological samples with three technical replications and at least two repetitions were used. Error bars indicate SEM.

Although our previous analyses did not support any strong direct links between differential cytosine methylation and senescence-associated gene expression, we performed qRT-PCR on the same leaf samples as those used for WGBS to ascertain the temporal relationships between cytosine methylation and gene expression for at least a few genes. The promoter region of *ROS1* (*AT2G36490*) appeared to be the most interesting because it was hypomethylated in the early stages of flowering, but methylation partially recovered at SM (Fig. 6A). The location of this transient DMR corresponded to a genomic position that was previously recognized as a DNA methylation monitoring system (MEMS) involved in gene methylation regulation via *ROS1* (Lei *et al.*, 2015; Zhang *et al.*, 2018). Less methylation in all three cytosine contexts was observed in the *ROS1* MEMS region (Fig. 6A) between Blt and Flwr, which coincided with reduced *ROS1* expression, as expected from the nature of this region (Lei *et al.*, 2015).

Another DMR found upstream in close proximity to the phytochrome *PHYC* gene spanned 16 nucleotides and occurred only in the CHH context. Less methylation was observed between Flwr and SD (Fig. 6B), coinciding with a small increase in *PHYC* expression. However, several senescence up-regulated genes, such as *AT3G44530* (encodes a WD-containing component of a putative HISTONE chaperone complex), *AT1G44820* (encodes a putative aminoacylase), *AT5G53120* (encodes a putative spermine synthase) and *AT1G66890* (unknown function) that were associated with massive cytosine methylation losses in their promoters in previous analyses during senescence (Yuan *et al.*, 2020), had more diverse methylation patterns in our study (Fig. 6C–F). For *AT3G44530*, a gene up-regulated at early stages, no changes in cytosine methylation were detected in our dataset. Methylation in the *AT1G44820* promoter increased transiently, while up-regulation of gene expression increased at early stages. The methylation in the *AT5G53120* promoter increased at early stages, whereas gene expression transiently increased until SD and then decreased. A slight reduction in *AT1G66890* expression levels until SD was associated with the loss of a cytosine methylation DMR in its promoter before flowering (Fig. 6F). Any simple, causal temporal relationship between methylation in gene promoters and senescence-associated gene expression regulation appears unlikely for this small set of genes.

### Cytosine methylation in bZIP and WRKY transcription factor binding sites

We therefore considered the possibility that DNA/gene transcription factor interaction networks as a whole might be affected by differential cytosine methylation at many unrelated loci. Cumulative effects within gene expression networks might then only be expected when several transcription factor binding sites are targeted by differential methylation in certain motifs, as in the case of the *ddc* mutant (Fig. 2E). Indeed, we found 2300 DMLs inside bZIP binding sites, of which 71 coincided with DMRs, and 3892 DMLs in W-boxes, of which only 25 matched with DMRs. Differentially methylated transcription factor binding sites were, however, only a minor fraction of genome-wide sites; they represented only 3% and 1.7% (of a total 73098 bZIP and 235305 WRKY sites), respectively.

**Fig. 7.**
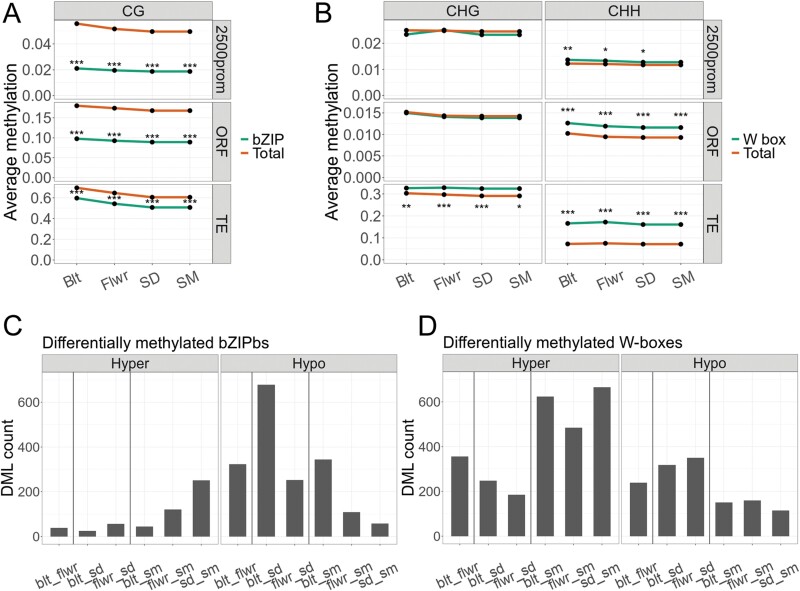
Average methylation levels of bZIP and WRKY transcription factor binding sites (green) compared with overall genome methylation (orange) in regions 2500 bp upstream of TSS, ORFs, and TEs in their respective cytosine context. (A) Average methylation of A-, C-, and G-boxes. (B) Average methylation of W-boxes. Bootstrap analysis was performed. Significance levels: *P<0.01; **P<0.001; ***P<0.0001. (C) Number of A-, C-, G-, or (D) W-boxes intersecting with DMLs.

We noted that the methylation of bona fide bZIP binding sites that contained A-, C-, or G-box palindromes with a CG motif was depleted in all genomic regions, in promoters, in ORFs and TEs. The largest differences were observed in promoter regions 2500 bp upstream of TSS (Fig. 7A). Despite the large number of differentially methylated bZIP sites, average temporal methylation changes at these sites were small and declined until the SD stage. In contrast, the conserved six-nucleotide sequence that is targeted by WRKY transcription factors (known as the W-box, *TTGACT/C*) had a different methylation status in TEs compared with the overall methylation status of cytosines in the same context in other regions (Fig. 7B), but again, the average temporal variation was small. The CHH context of the W-box (*TTGA****C****T****H*** and *TTGAC****CHH***) had a significantly higher methylation level in promoters, ORFs, and TEs, but the overall occurrence of CHH methylation in W-boxes in 2500 bp promoters was the lowest. These differences were most significant at Blt and the statistical significance progressively declined during senescence (Fig. 7B). ORFs and TEs maintained higher methylation throughout the time course. The CHG context appeared less often in a W-box (*TTGA****CTG*** and *TTGA****CCG***) and had higher methylation only in TEs. Even though the two groups of transcription factor binding sites exhibited opposite behaviour in terms of cytosine methylation, their presence in or close to ORFs was consistently associated with lower methylation levels, compared with other genomic regions.

In total, 358 genes were found with differentially methylated A-, C-, or G-boxes (Supplementary Table S5). Significant over-representation was found in only one group, comprising seven genes related to the regulation of gamete fertilization, all of them coding for ECA1 gametogenesis-related family proteins. Interestingly, a differentially methylated A-box was located in the vicinity of the *PHYC* gene (Fig. 6B), together with a differentially methylated G-box near *NAC019*. Five hundred and seventeen genes were found with a differentially methylated W-box in their proximity (Supplementary Table S6), but no significant over-representation (and no relationship with senescence-associated genes) was found in this group. Notable genes were *WRKY74* with three differentially methylated W-boxes, and *WRKY41* with two differentially methylated W-boxes. *WRKY64*, *NAC100*, *NAC003*, and *RDR1* contained one differentially methylated W-box. Even though none of these genes are recognized as major regulators of senescence, this list agrees with the potential involvement of differential methylation in transcription factor binding networks.

## Discussion

The involvement of epigenetic regulatory mechanisms in plant longevity, leaf ageing, and senescence has been previously established (Ay *et al*., 2009, 2014). Here, we initially tested whether the aberrant maintenance of cytosine methylation in the *ddc* and *ros1* mutants resulted in senescence- and remobilization-specific phenotypes. Clearly, these mutants have pleiotropic developmental defects (with flowering initially reported to be unaffected by non-CG methylation; Chan *et al.*, 2006) and we cannot exclude that methylation changes are secondary. However, in our conditions the hypomethylated *ddc* mutant flowered later than *ros1* and had delayed first symptoms of senescence compared with hypermethylated *ros1* (Fig. 1). The execution of the senescence programme following the appearance of the first symptoms tended to be faster in both mutant lines compared with the wild type and resulted in altered leaf to seed ratio and N remobilization from the rosette and an overall higher C:N ratio at the end of the process. Diaz *et al.* (2008) found similar N partitioning between sink and source tissues, but this did not correlate with early or late senescence phenotypes. Clearly, methylation mutants have pleiotropic phenotypes and cytosine methylation might indirectly alter the C:N ratio or hormonal regulation. Indeed, increased IAA concentrations were observed in the leaves of *ddc* seedlings, consistent with differences in the expression of genes involved in auxin synthesis, transport, and signalling (Forgione *et al.*, 2019).

Furthermore, proper maintenance of cytosine methylation was required during N limitation. While transient N withdrawal caused significant reductions in rosette biomass in all three genotypes, the two mutants did not completely recover transiently after N resupply and showed lower terminal N concentrations in the rosette compared with Col-0 (Fig. 2). In accordance with the findings of Lin and Tsay (2017), N limitation delayed flowering in Col-0, but this was not seen in the methylation mutants (Fig. 2). Furthermore, both mutants apparently more efficiently remobilized N to seeds, which might be interesting from an agronomical point of view, if confirmed in crops. *ros1* produced more seed biomass, regardless of the treatment. Lower leaf to seed ratios, together with higher N remobilization and higher C:N ratios in the rosette under control conditions agree with faster senescence progression and differential N remobilization in *ddc* and *ros1* (Figs 1, 2). Interestingly, Kuhlmann *et al.* (2020) have suggested an involvement of cytosine methylation in the regulation of specific loci related to shoot growth under N deficiency in Arabidopsis. Cytosine methylation changes associated with leaf senescence, however, did not specifically target N-related genes or their promoters in our experiments (Supplementary Tables S3–S6). Nevertheless, several *NAC* and *WRKY* transcription factor genes including one involved in senescence, namely *NAC019* (Hickman *et al.*, 2013; Takasaki *et al.*, 2015), were differentially methylated, implying that these might gradually orchestrate changes in transcription factor networks.

We chose four physiologically relevant stages and five distinct leaves per plant for various analyses to relate these to ongoing leaf senescence. Colour, size, and weight correlated with chlorophyll concentrations (Fig. 3; Supplementary Fig. S6). In close agreement with previous reports, net loss in chlorophyll concentration (due to repression of its biosynthesis starting around the induction of flowering at 21 d after seeding; Breeze *et al.*, 2011) occurred as soon as the transition of the shoot apical meristem to reproductive growth occurred. This was accompanied by increases in soluble sugar content (Wingler *et al.*, 2006), elevated H_2_O_2_ levels, and a moderate overall reduction in cytosine methylation (Fig. 4). Comparing leaf material at certain stages (instead of sampling at fixed days after seeding) decreased the variance within sampling groups, because the senescence time course of individual plants is subject to substantial variation even in a single genotype. Differences in flower induction of the two methylation mutants (Figs 1, 2) are likely explained by distinct cytosine methylation of crucial genes and their promoters (Finnegan *et al.*, 2005; Kinoshita *et al.*, 2007; Zicola *et al.*, 2019). Hormonal changes during leaf senescence appeared later in the process (Fig. 3; Supplementary Fig. S6) and identified a strong activation of defence reactions. Increases in SA and phytoalexins at SD preceded elevation of JA and ABA. It is interesting to note that widespread DNA methylation changes are associated with biotic stress responses (Dowen *et al.*, 2012), and that such stress responses were prominent at the terminal stages, at which *de novo* methylation occurred (Fig. 4).

We found moderate methylome changes and an interesting discrepancy between differential methylation in CG, CHG, and CHH contexts; more than 50% of all hypomethylated loci appeared in the CG context (Figs 4, 5). This trend shifted towards CHG and CHH between SD and SM. On the other hand, hypermethylation occurred predominantly in CHG and CHH contexts, with a slight shift towards CG during SM. Previous studies suggested that the two main methyltransferase genes involved in CG methylation maintenance, namely *MET1* and *CMT3*, are down-regulated after flower induction in Arabidopsis (Breeze *et al.*, 2011; Ogneva *et al.*, 2016). Leaf growth ceased around Flwr, so the hypomethylation in CG is at least partially attributed to the reduced maintenance of methylation following genome duplication, as leaf cells in Arabidopsis divide almost until the final leaf size is reached, with endoreduplication occurring from the time that cell division rates decline until the end of cell expansion. The relatively low number of DMRs is clearly also due to the fact that even at the defined sampling times, leaf cells are visibly in different stages within the same leaf. Clearly, senescence progression from the tip is not entirely uniform in every leaf (Fig. 3A, C) and it might be necessary to resolve stage-dependent methylomes at the cellular level. Our 25% criterion for DMLs and DMRs was chosen to account for discrepancies between such cell stage differences in the same leaf; differential methylation at 25% likely reflects a 100% change in the methylation of a quarter of all leaf cells (as methylation is either present or not). It may be possible (and necessary) in the future to separate different cellular leaf senescence stages by cell sorting of enzymatically isolated leaf cell protoplasts. Still, hypomethylation was clearly not concentrated in specific genomic regions, but rather appeared throughout the genome. The partial recovery of CG methylation after the cessation of leaf expansion, between SD and SM, might be the result of residual activity of maintenance methyltransferases. However, the large number of hypomethylated loci when Blt is compared with SM indicates that this increase is caused by *de novo* methylation, rather than the recovery of lost methyl-cytosines. Accordingly, the main methyltransferase genes involved in CHG and CHH methylation, namely *DRM1* and *DRM2*, showed (mostly) stable expression during senescence (Breeze *et al.*, 2011; Ogneva *et al.*, 2016). This suggests a functional role of RdDM and *de novo* methylation in leaf senescence progression as its loss affects senescence progression and agrees with our finding that the triple mutant *ddc* has an aberrant senescence and remobilization phenotype (Figs 1, 2). This is further supported by the finding that loss-of-function mutants of important components of the RdDM pathway, *DEFECTIVE IN RNA-DIRECTED DNA METHYLATION1* (*DRD1*) and *DECREASED DNA METHYLATION1* (*DDM1*), had delayed senescence (Cho *et al.*, 2016). These mutants also had an altered DNA methylation profile (Stroud *et al.*, 2013), further pointing to the involvement of the RdDM pathway and methylome dynamics in senescence.

Interestingly, dark-induced leaf senescence, a process more similar to the ultimate senescence steps between SD and SM, was associated with moderate hypomethylation in the CHH context (Trejo-Arellano *et al.*, 2020), demonstrating differences between the faster, stress-induced senescence and the slowly progressing age-related type of senescence studied here. Similar to our study, the methylation changes in the CHH context in dark-induced senescence did not correlate with senescence-associated gene expression (but were associated with the up-regulation of young transposons; Trejo-Arellano *et al.*, 2020). In agreement with that study, the ultimate stages in our experimental set-up also consistently targeted predominantly the CHH context, in agreement with ongoing RdDM. In contrast, Yuan *et al.* (2020) have described similar patterns of CG demethylation during initial phases of natural senescence in leaves, as observed here (Fig. 3), but did not observe the hypermethylation of CHH and CHG at late stages, probably because their analysis relied on fewer and earlier time points during senescence progression. It is likely that discrepancies between the WGBS analysis in our study and that of Yuan *et al.* (2020) are partially due to differences in growth conditions, physiological states, bioinformatic analyses, and sample sizes. We used three biological replicates comprising pooled samples from ~20 plants in total, followed by a procedure based on a Bayesian hierarchical model utilized by the DSS package from bioconductor for DML and DMR scoring, whereas Yuan *et al.* (2020) used two biological replicates and Fisher’s exact test for DMR discovery, without explicit counting of DMLs. Furthermore, their general analysis compared methylation levels at 100 kb windows, whereas our analysis was based on individual cytosines. Finally, our experiment proceeded beyond the 50% yellowing of the leaves defined as their last time point and at that later stage (SM), we observed the highest degrees of CHH hypermethylation. Still, the proposed concerted methylation loss particularly in senescence-associated genes or their promoters, as well as the proposed delay of senescence by hypermethylation (Yuan *et al.*, 2020), are not supported by our experimental set-up.

Despite that individual methylated cytosines may have profound effects on transcription factor binding to methylated DNA (O’Malley *et al.*, 2016), only accumulations of differentially methylated cytosines in genomic regions are generally considered genetically relevant (Quadrana and Colot, 2016). Biochemical binding assays to methylated WRKY target sites strongly suggested that WRKYs’ ability to bind their target sites is strongly prohibited by methylation, but it is important to note that we used only fully methylated targets in our binding assays (Fig. 2G; Supplementary Fig. S4). While methylation within the chromosomal DNA of a cell is either present or not, gradually smaller methylation changes in a tissue suggest that target binding is affected in fewer cells, with limited effect on average gene expression within a tissue. Strong effects of methylation on target binding are supported by independent experiments (O’Malley *et al.*, 2016).

To find genes potentially regulated via cytosine methylation, we searched for DMRs located closely to their transcription start sites (Fig. 5). Cytosine methylation around the TSS and in promoter regions has long been known to be involved in the regulation of gene expression (Finnegan *et al.*, 1996; Jeddeloh *et al.*, 1998; Wang *et al.*, 2004). Interestingly, we have found a hypomethylated DMR lying in close proximity to the TSS of the demethylase gene *ROS1* (Fig. 6). Lei *et al.* (2015) have described a 39 bp DNA MEMS found between the TSS of *ROS1* and an adjacent helitron TE. Low methylation levels of this region are associated with the reduced expression of *ROS1*, whereas high methylation levels correlate with a higher expression (Lei *et al.*, 2015; Fig. 6). The decline of *ROS1* expression with the progression of senescence suggests reduced demethylation activity and strengthens the idea that the loss of methylation during senescence is attributable to inhibited maintenance, rather than to active demethylation (Figs 1, 2).

The cytosine methylation changes during progressing leaf senescence were not preferentially associated with any physiological gene class, e.g. with nutrient status, defence- or senescence-associated genes. For the key regulatory gene *WRKY53*, no changes in its cytosine methylation have been observed, despite its upregulation during early senescence (Zentgraf *et al.*, 2010). Because high-resolution transcriptomics datasets of progressing senescence exist (e.g. Breeze *et al.*, 2011; Yuan *et al.*, 2020) we did not obtain another transcriptome dataset, but concentrated just on a few genes. We confirmed a CHH DMR in the 2000 bp region adjacent to the *PHYC* gene (Fig. 6; Yuan *et al.*, 2020) and a differentially methylated A-box in its promoter. Changes in *PHYC* expression were correlated with this DMR, but the temporal inspection of other senescence-associated genes such as those identified in Yuan *et al.* (2020) failed to support the simple conclusion that DMRs and senescence-associated gene expression were causally or temporally linked (Fig. 6; Yuan *et al.*, 2020). Many other recent studies also have failed to identify a general, simple direct relationship between methylation changes in tissues and gene expression changes (Quadrana and Colot, 2016; Zhang *et al.*, 2018). Other epigenetic factors, such as the link with chromatin (de-)condensation or histone marks, are probably involved.

Aberrant methylation in transcription factor binding sites may have caused disturbances in signal transduction networks during senescence, e.g. of the WRKY53, WRKY18, and WRKY25 interaction network (Zentgraf and Doll, 2019). WRKY transcription factors preferentially bind the *cis*-element *TTGACT/C*, named a W-box, which contains cytosines in CHH or, in some cases, CHG contexts. Full methylation of target sites impairs WRKY binding *in vitro* (Fig. 2; Supplementary Fig. S4). By an independent and different approach, O’Malley *et al.* (2016) have demonstrated that DNA binding of more than 75% of transcription factors in Arabidopsis is sensitive to methylation, including WRKY18 and WRKY25. Methylation in W-boxes (in the CHH context) changed between stages, and this happened most strongly in TEs, followed by ORFs. W-boxes in regions adjacent to the TSS of ORFs were least methylated (Fig. 7). We speculate that cytosine methylation in genomic regions other than promoters serves to redirect transcription factors from unwanted non-functional sites. Therefore, the disruption of methylation maintenance in CHH and CHG contexts in the *ddc* mutant might indirectly affect the concentration of free unbound WRKY transcription factors and ultimately affect complex signalling networks. A link to aberrant WRKY binding in the *ros1* mutant is also supported by the finding that ROS1 erases methylation in certain promoters with WRKY binding sites (Halter *et al.*, 2021). A-, C-, and G-boxes also have the lowest methylation when in close proximity to transcription start sites, imposing limited access of methyltransferases to these regions, resulting in lower methylation levels. Any Arabidopsis gene has been estimated to be subjected to an average of 25–75 TF binding events and most of these are expected to have little effect on gene expression (Ferrier *et al.*, 2011; Jones and Vandepoele, 2020). As approximately 5% of genes in Arabidopsis are methylated in promoter regions, we therefore expect for these a complex cumulative relationship of individual DMLs and DMRs with gene expression.

**Fig. 8.**
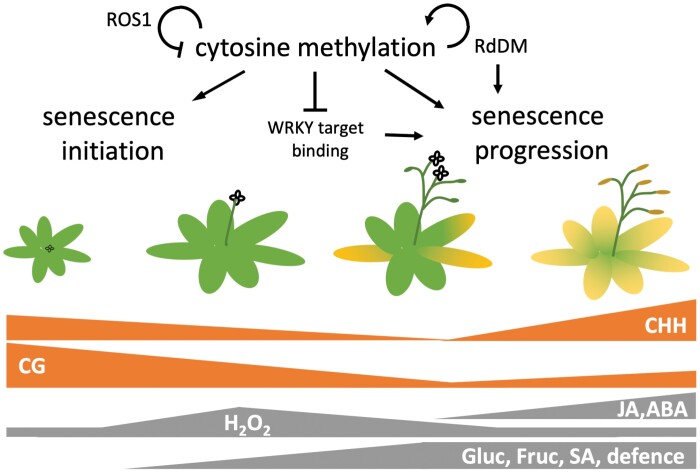
Relationship between flowering, senescence, and methylation. Changes in physiological indicators and cytosine methylation during the senescence process at the four time points, namely bolting, flowering, seed development and seed maturation, are given. ABA: abscisic acid; CHH, CG: context specific methylation; Fruc: fructose; Gluc: glucose; H_2_O_2_: hydrogen peroxide; JA: jasmonic acid; SA: salicylic acid.

We present a schematic working model summarizing the observed links of cytosine methylation with senescence progression and generative growth (Fig. 8). The quicker senescence progression and N remobilization in the *ddc* and *ros1* mutants is related to altered C:N ratios and may be secondary. The majority of cytosine methylation remains remarkably stable, although overall cytosine methylation transiently decreased during the initial phase (Fig. 4). The majority of hypermethylated loci at the ultimate time point was in CHH contexts, pointing to the involvement of functional RdDM (Cho *et al.*, 2016; K. Humbeck, personal communication) and potentially involves the DRM1 and DRM2 methyltransferases. Initial hypomethylation has been observed predominantly in CG contexts, indicating the inhibition of the maintenance of methylation primarily at earlier stages. The regulation of *ROS1* via differential methylation at its own promoter region early after flower induction (Fig. 6) indicates a possible downregulation of active demethylation, which is missing in *ros1* mutants. Despite that the DNA methylation pattern changes with senescence progression in the leaf, we consider any direct effect on the expression of yet known key senescence-associated genes unlikely.

Our results indicate that moderate methylation loss and the establishment of *de novo* methylation are associated with execution of the leaf senescence programme and nitrogen remobilization, which is altered in methylation mutants.

## Supplementary data

The following supplementary data are available at *JXB* online.

Fig. S1. Stable expression of the reference gene *ACTIN2* under all experimental conditions.

Fig. S2. Visual appearance of the leaf rosette at transition to flowering.

Fig. S3. Analyses of cytosine methylation data of *ddc* and Col-0 based on methylome data from Stroud *et al.* (2013).

Fig. S4. Influence of cytosine methylation on the binding of WRKY25 and WRKY18 via DPI-ELISA.

Fig. S5. Four developmental stages were analysed during the experiment.

Fig. S6. Physiological determinants of senescence in leaves numbered 5–9 from Col-0 plants.

Fig. S7. Change of defence-related secondary metabolites in leaf no. 7.

Fig. S8. Distribution of differentially methylated cytosines in CG, CHG, and CHH contexts along chromosome 1 in three pairwise comparisons.

Table S1. Primers used.

Table S2. Statistics of methylome sequencing.

Table S3. Gene list of ORFs intersecting with DMRs.

Table S4. Gene list of 2500 promoters intersecting with DMRs.

Table S5. Genes/promoters with differentially methylated A-, C-, or G-boxes.

Table S6. Genes/promoters with differentially methylated W-boxes.

erac167_suppl_Supplementary_Figures_S1-S8

erac167_suppl_Supplementary_Tables_S1-S6

## Data Availability

The data that support the findings of this study are openly available in the Gene Expression Omnibus (GEO) database under the accession number GSE176468.

## Abbreviations

ABA: abscisic acid
Blt: bolting
C: cytosine
C:N ratio: carbon:nitrogen ratio
DAS: days after sowing
DML: differentially methylated locus
DMR: differentially methylated region
Flwr: flowering
G: guanine
H: any base of A, T, C
JA: jasmonic acid
ORF: open reading frame
PCA: principle component analysis
RdDM: RNA-directed DNA methylation
ROS: reactive oxygen species
ROS1: repressor of silencing1
SA: salicylic acid
SD: seed development
SM: seed maturation
TE: transposable element
TF: transcription factor
TSS: transcription start site
WGBS: whole genome bisulfite sequencing
