# Supplementary material for: Moderate DNA methylation changes associated with nitrogen remobilization and leaf senescence in Arabidopsis
Source: J Exp Bot. 2022 May 13;73(14):4733–52. doi: 10.1093/jxb/erac167 (PMC9366325; doi:10.1093/jxb/erac167)
Supplement: erac167_suppl_Supplementary_Figures_S1-S8 [file erac167_suppl_supplementary_figures_s1-s8.pdf]

Suppl. dataset 1 (Suppl. figures with legends)

For: **Moderate DNA methylation changes associated with nitrogen remobilization and leaf senescence in *Arabidopsis***

By Emil Vatov et al.

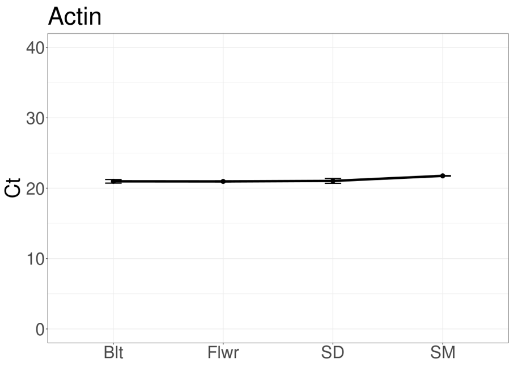

Suppl. Figure S1. Stable expression of the reference gene *ACTIN2* under all experimental conditions.

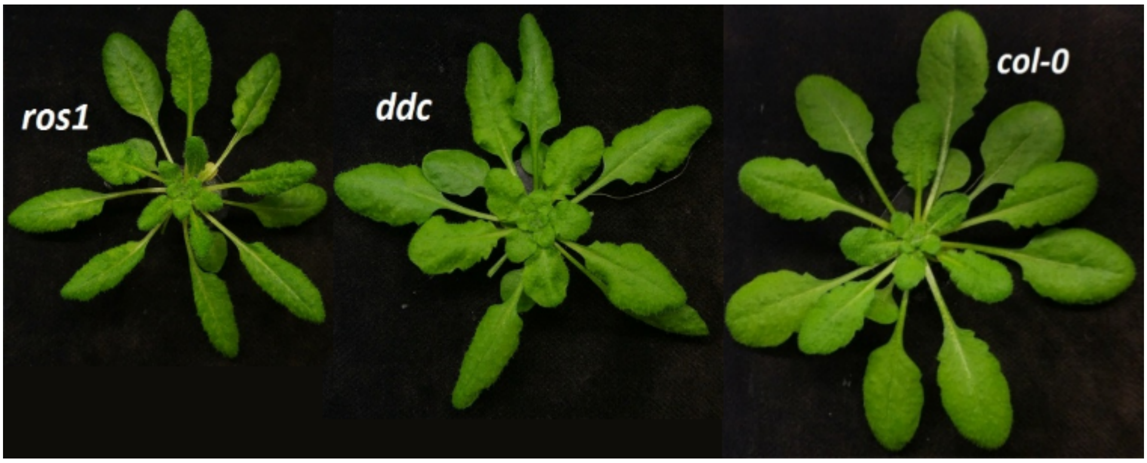

Suppl. Figure S2. Visual appearance of the leaf rosette at transition to flowering. From left to right: Col-0, *ddc* and *ros1.* Note that *ros1,* but not the other two genotypes, already has one senescent leaf.

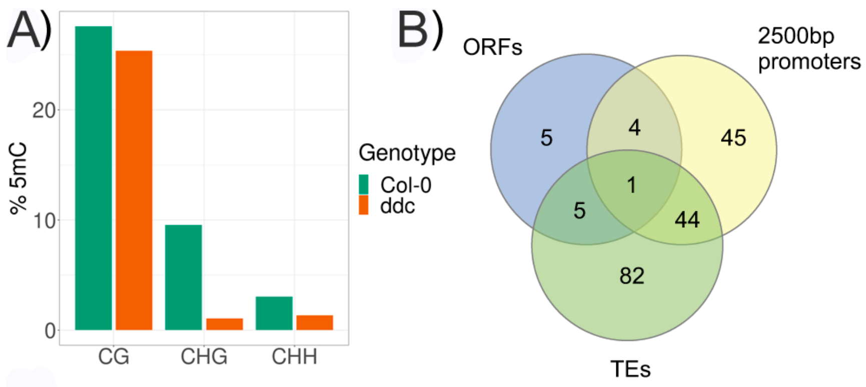

Suppl. Figure S3. Analyses of cytosine methylation data of *ddc* and Col-0 based on methylome data from Stroud et al., (2013). (A) Cytosine methylation levels in CG, CHG and CHH contexts. (B) Number of DMRs (out of a total of 228) intersecting with ORFs, 2500bp promoters regions and TEs.

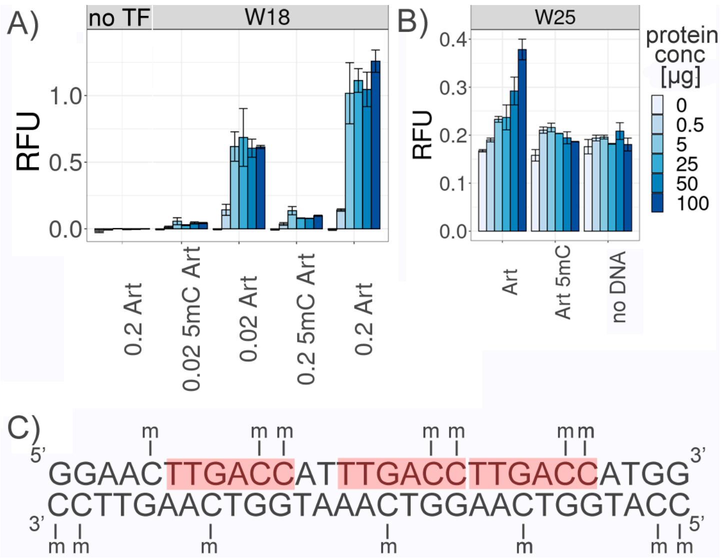

Suppl. Figure S4. Influence of cytosine methylation on the binding of WRKY25 and WRKY18 via DPI-ELISA. (A) Affinity of WRKY18 to an artificial promoter sequence with or without cytosine methylation. 0.2 and 0.02 Art indicate 20 pmol and 2 pmol of double-stranded artificial promoter DNA fragments per 60 µl, respectively. 5mC indicates methylated DNA. (B) Affinity of WRKY25 to an artificial promoter sequence with (5mC) or without cytosine methylation. Colour codes indicate the concentrations of protein (conc) used in µg per 60 µl reaction. (C) Double-stranded sequence of the artificial (Art) methylated promoter (5mC). W-boxes are highlighted in red colour. Each experiment was carried out with 2 technical replicates and was repeated at least twice.

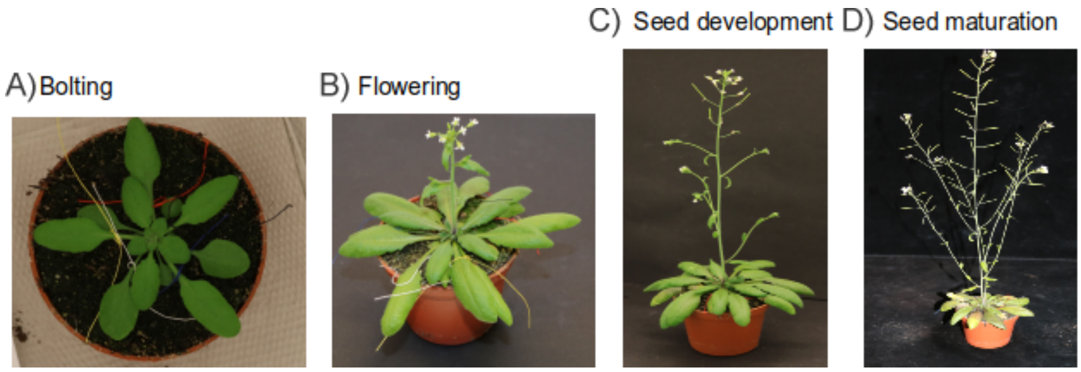

Suppl. Figure S5. Four developmental stages were analysed during the experiment: (A) Bolting, (B) Flowering, (C) Seed development and (D) Seed maturation.

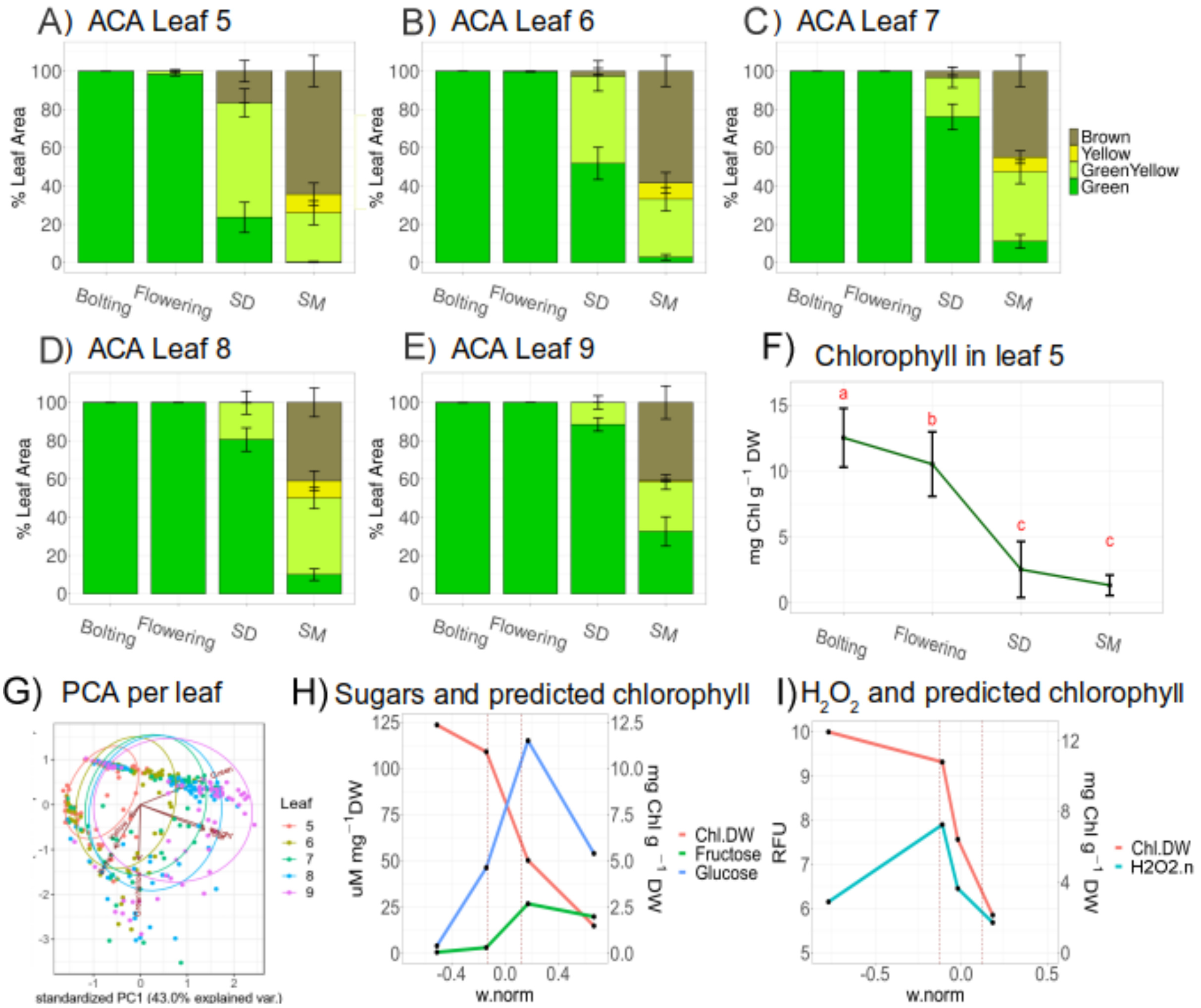

Suppl. Figure S6. Visual quantification of senescence in leaves numbered 5 to 9 of Col-0 plants. Automated colorimetric assay (ACA) was performed on pixel counts for green, green-yellow, yellow and brown coloration, total pixel count and leaf fresh weight (n = 20 $\pm$ SEM). (A) Leaf 5. (B) Leaf 6. (C) Leaf 7. (D) Leaf 8. (E) Leaf 9. (F) Chlorophyll in leaf 5 (n = 20 $\pm$ SD). (G) PCA for distinguishing the colours of the various leaves. (H) Glucose and fructose per predicted chlorophyll concentrations. (I) Hydrogen peroxide vs. chlorophyll concentrations plotted against normalized leaf weight. w.norm = normalized leaf weight on a scale of -1 to 1, where -1 to 0 indicates leaf growth and 0 to 1 indicates leaf desiccation and loss of weight. Chl.DW = predicted chlorophyll concentration in leaf dry weight

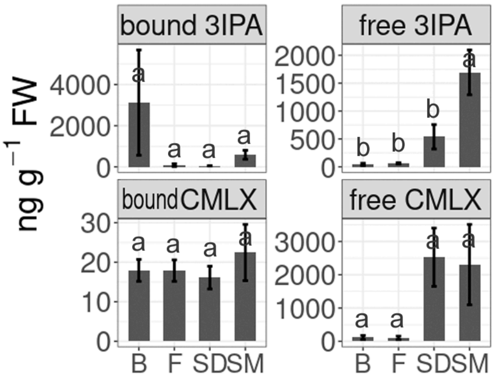

Suppl. Figure S7. Change of auxin precursor 3IPA and defence-related phytoalexins in bound and free form in leaf number 7, at four time points measured in arbitrary units (A.U.; n = 3; means ± SD).

A)

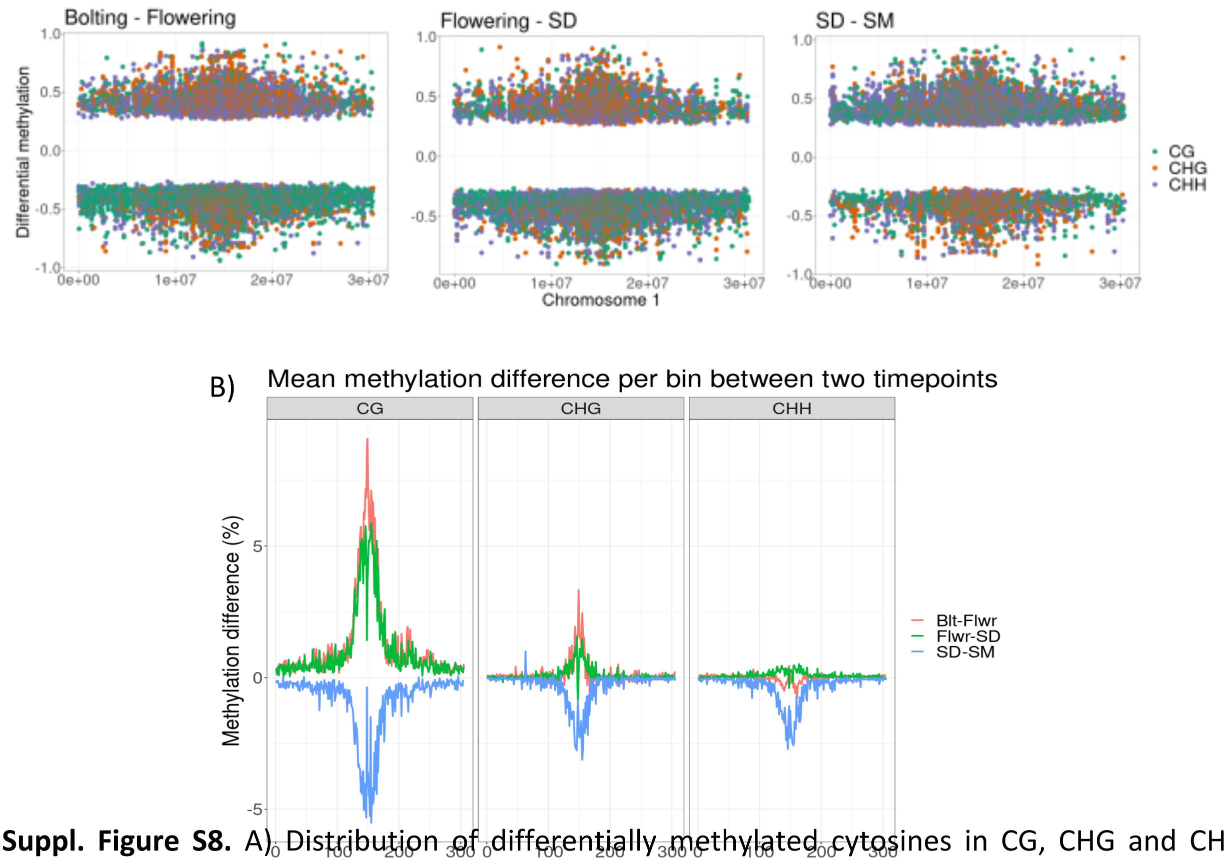

Suppl. Figure S8. A) Distribution of differentially methylated cytosines in CG, CHG and CHH contexts along chromosome 1 in three pairwise comparisons. B) Difference in mean methylation between two pairwise comparisons calculated per 100 000 bp bins along chromosome 1 in CG, CHG and CHH contexts.
